# Chromatin-Remodelling Complex NURF Is Essential for Differentiation of Adult Melanocyte Stem Cells

**DOI:** 10.1371/journal.pgen.1005555

**Published:** 2015-10-06

**Authors:** Dana Koludrovic, Patrick Laurette, Thomas Strub, Céline Keime, Madeleine Le Coz, Sebastien Coassolo, Gabrielle Mengus, Lionel Larue, Irwin Davidson

**Affiliations:** 1 Department of Functional Genomics and Cancer, Institut de Génétique et de Biologie Moléculaire et Cellulaire, CNRS/INSERM/ULP, Illkirch, France; 2 Beaston Institute for Cancer Research, Glasgow, United Kingdom; 3 Department of Oncological Sciences, Icahn School of Medicine at Mount Sinai, New York, New York, United States of America; 4 Institut Curie CNRS UMR3347, INSERM U1021, Bat 110, Orsay, France; 5 Equipes labélisées Ligue Contre le Cancer, Orsay and Strasbourg, France; Medical Research Council Human Genetics Unit, UNITED KINGDOM

## Abstract

MIcrophthalmia-associated Transcription Factor (MITF) regulates melanocyte and melanoma physiology. We show that MITF associates the NURF chromatin-remodelling factor in melanoma cells. ShRNA-mediated silencing of the NURF subunit BPTF revealed its essential role in several melanoma cell lines and in untransformed melanocytes *in vitro*. Comparative RNA-seq shows that MITF and BPTF co-regulate overlapping gene expression programs in cell lines *in vitro*. Somatic and specific inactivation of *Bptf* in developing murine melanoblasts *in vivo* shows that *Bptf* regulates their proliferation, migration and morphology. Once born, Bptf-mutant mice display premature greying where the second post-natal coat is white. This second coat is normally pigmented by differentiated melanocytes derived from the adult melanocyte stem cell (MSC) population that is stimulated to proliferate and differentiate at anagen. An MSC population is established and maintained throughout the life of the Bptf-mutant mice, but these MSCs are abnormal and at anagen, give rise to reduced numbers of transient amplifying cells (TACs) that do not express melanocyte markers and fail to differentiate into mature melanin producing melanocytes. MSCs display a transcriptionally repressed chromatin state and Bptf is essential for reactivation of the melanocyte gene expression program at anagen, the subsequent normal proliferation of TACs and their differentiation into mature melanocytes.

## Author Summary

The melanocytes pigmenting the coat of adult mice derive from the melanocyte stem cell population residing in the permanent bulge area of the hair follicle. At each angen phase, melanocyte stem cells are stimulated to generate proliferative transient amplifying cells that migrate to the bulb of the follicle where they differentiate into mature melanin producing melanocytes, a processes involving MIcrophthalmia-associated Transcription Factor (MITF) the master regulator of the melanocyte lineage. We show that MITF associates with the NURF chromatin-remodelling factor in melanoma cells. NURF acts downstream of MITF in melanocytes and melanoma cells co-regulating gene expression *in vitro*. *In vivo*, mice lacking the NURF subunit Bptf in the melanocyte lineage show premature greying as they are unable to generate mature melanocytes from the adult stem cell population. We find that the melanocyte stem cells from these animals are abnormal and that once they are stimulated at anagen, Bptf is required to ensure the expression of melanocyte markers and their differentiation into mature adult melanocytes. Chromatin remodelling by NURF therefore appears to be essential for the transition of the transcriptionally quiescent stem cell to the differentiated state.

## Introduction

MIcrophthalmia-associated Transcription Factor (MITF) is a basic helix-loop-helix leucine zipper (bHLH-Zip) factor playing an essential role in the differentiation, survival, and proliferation of normal melanocytes, and in controlling the melanoma cell physiology [1–4]. Quiescent murine adult melanocyte stem cells (MSCs) residing in the bulge region of the hair follicle do not express MITF, but its expression is induced in the proliferating transit amplifying cells (TACs) that are generated at anagen [5–7]. MITF expression persists as TACs migrate towards the bulb to form terminally differentiated melanin producing melanocytes [8,9].

Following malignant transformation of melanocytes, cells expressing low or no MITF are slow cycling and invasive, displaying enhanced tumour initiating properties, high MITF activity is characteristic of proliferative melanoma cells, and even higher MITF activity is associated with terminal differentiation of melanocytes [10]. MITF silencing in proliferative melanoma cells leads to cell cycle arrest and entry into senescence [11,12]. These and other observations gave rise to the proposed ‘rheostat’ model postulating that the level of functional MITF expression determines many biological properties of melanocytes and melanoma cells [13,14]. MSCs and slow cycling melanoma cells express low or no MITF, while TACs and proliferative melanoma cells express higher levels. High MITF activity induces terminal melanocyte differentiation and can also induce cell cycle arrest of melanoma cells [15]. This property has been exploited to derive drugs that induce terminal differentiation of melanoma cells as a therapy for melanoma [16].

MITF is both an activator and a repressor of transcription and functions through a host of cofactors. A comprehensive analysis of the MITF ‘interactome’ in 501Mel melanoma cells revealed that the NURF (Nucleosome Remodelling Factor) complex associates with MITF [17]. NURF was first identified in *Drosophila Melanogaster* [18,19] and comprises NURF301 (in mammals, BPTF, Bromodomain, PHD-finger Transcription Factor), the ISWI-related SNF2L (SMARCA1) ATPase subunit, NURF55 (RbAp46, RBBP7) and NURF38 [20–23]. NURF promotes ATP-dependent nucleosome sliding and transcription from chromatin templates *in vitro* [24–26]. Mammalian NURF comprises BPTF, RBBP4 and SNF2L and may further comprise the SNF2H (SMARCA5) ATPase subunit, BAP18 and HMG2L1 [27,28]. The 450 kDa BPTF is the defining and only unique subunit of NURF and binds active promoters via the interaction of its PHD (plant homeodomain) domain with trimethylated H3K4 and of its bromodomain with acetylated H4K16 [21,29,30].

Despite extensive characterisation of the biochemical properties of the NURF complex and its BPTF subunit [31], much less is known about their biological functions in mammals. *Bptf* inactivation in mouse leads to embryonic lethality shortly after implantation [32,33]. Bptf loss is not however cellular lethal as it is possible to isolate viable *Bptf* ^-/-^ ES cells, but *in vitro* they show defective differentiation into mesoderm, and endoderm lineages [33]. Somatic inactivation of Bptf in CD4-CD8 double negative thymocytes has shown that it is required for their subsequent maturation [34]. Furthermore, BPTF may also be involved in maintaining human epidermal keratinocyte stem cells in an undifferentiated state *in vitro* [35].

The interaction of MITF with NURF prompted us to investigate its role in melanoma cells and in the melanocyte lineage. BPTF is selectively required in melanoma cells and melanocytes *in vitro*. Inactivation of *Bptf* in developing melanoblasts (*Bptf* ^mel-/-^) shows that it acts during their embryonic proliferation and migration, and we observed a unique phenotype in neonatal mice where the second post-natal anagen coat is devoid of pigment resulting in rapid loss of pigmentation and a lasting white pelage. Contrary to previous models where premature post-natal greying results from loss of MSCs, in *Bptf* ^mel-/-^ mice an MSC population persisted throughout the life of the animal, but at anagen, Bptf is required for normal TAC proliferation, for expression of melanocyte markers and hence for terminally differentiation into mature melanin producing melanocytes.

## Results

### MITF associates with NURF in melanoma cells

We previously described tandem affinity purification of N-terminal FLAG-HA epitope tagged MITF from the soluble nuclear and chromatin associated fractions of 501Mel cells [17]. Amongst the identified proteins were the BPTF, SMARCA5, SMARCA1 and RBBP4 subunits of the NURF complex. Multiple peptides for these proteins were detected in the chromatin-associated fraction from the cells expressing tagged MITF, whereas no peptides for these factors were found in immunoprecipitations from the control extract (S1A Fig). Interaction of these NURF components with MITF was confirmed in western blot experiments showing that BPTF, SMARCA1 and SMARCA5 all specifically precipitated with tagged MITF in the chromatin-associated fraction (S1B Fig). MITF therefore associates, either directly or indirectly, with the NURF complex on chromatin in 501Mel cells.

We next interrogated transcriptome data [36] to assess expression of the BPTF, SMARCA1 and SMARCA5 subunits of NURF in a collection of human melanoma cells. The three subunits were expressed at comparable levels in all of the tested cell lines, whether they expressed high (501Mel, 888-mel) low (SK-Mel-28, LYSE) or no (1205Lu, WM852) MITF (S1C Fig). The expression of BPTF, SMARCA1 and SMARCA5 proteins was also assessed in extracts from a subset of these lines. Again, the levels of each protein were comparable in the different lines (S1D Fig). NURF is therefore present in all types of melanoma cells irrespective of MITF expression and their tumourigenic properties.

### Selective requirement of BPTF in melanoma cells

**Fig 1:**
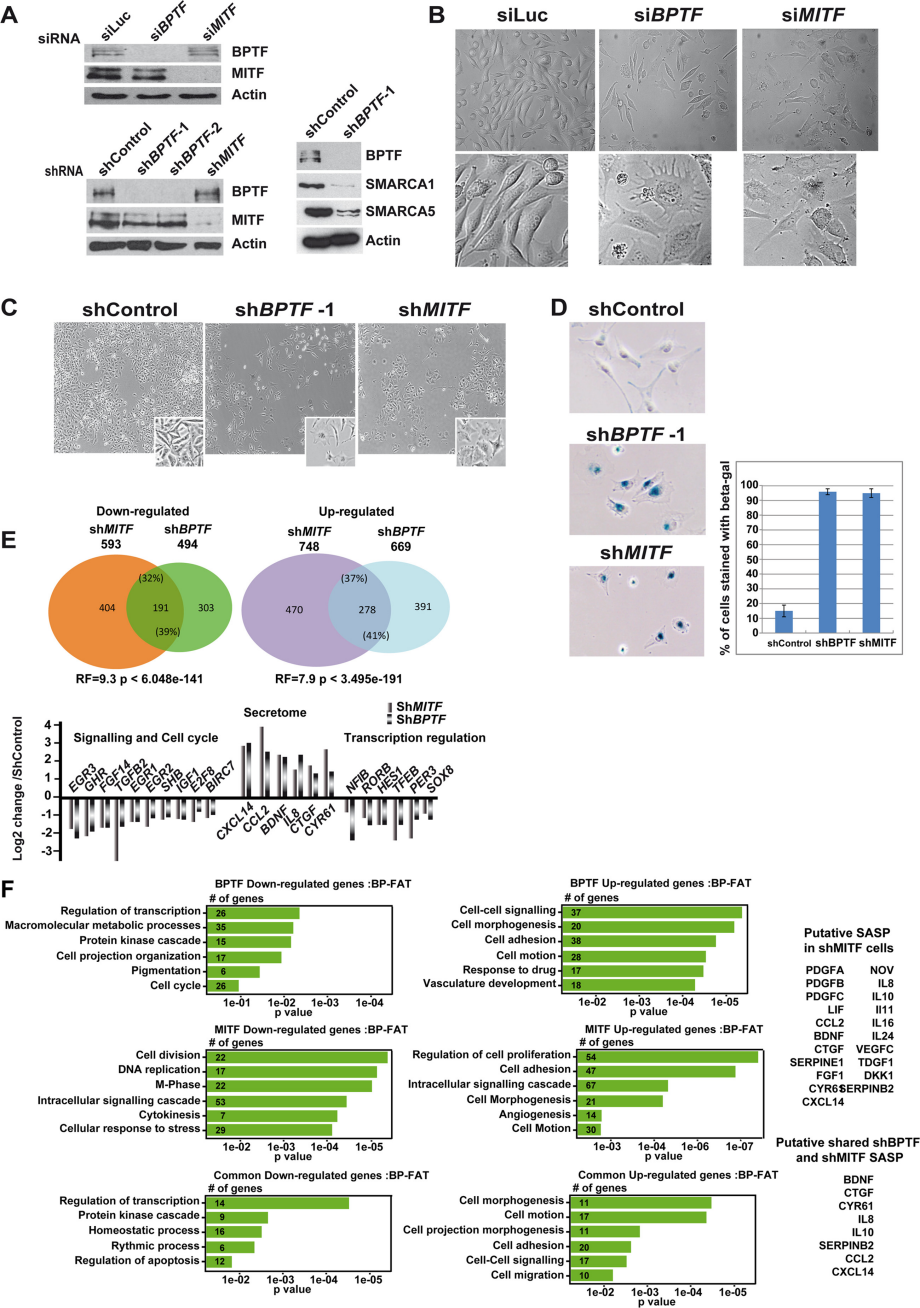
BPTF is essential in 501Mel cells. **A.** Western blots showing si/shRNA knockdowns of BPTF and MITF in 501Mel cells. **B**. Phase contrast microscopy of 501Mel cells following siBPTF and siMITF knockdown. Magnification X20. Inlays show enlargements (X40) of representative cells. **C.** Phase contrast microscopy of 501Mel cells following shBPTF and shMITF knockdown. Inlays show enlargements of representative cells. For simplification only shBPTF-1 cells are shown, but analogous images were made for shBPTF-2. **D.** Staining and quantification of 501Mel cells following shBPTF and shMITF knockdown for senescence-associated β-galactosidase. **E**. Venn diagrams illustrate the overlap between up and down-regulated genes following shBPTF and shMITF knockdown. Several examples of commonly regulated up and down-regulated genes are indicated based on the results of RNA-seq. RF shows the representation factor and p value for the overlaps between the up and down-regulated genes sets. **F** Ontology of genes up and down-regulated by shBPTF and shMITF knockdown and of commonly regulated genes. The number of genes in each category is indicated along with the p value. SASP components whose expression is induced by shMITF and ShBPTF are listed on the right.

To address the function of BPTF in 501Mel cells, we performed both siRNA and shRNA knockdown experiments (Fig 1A). SiBPTF led to prominent morphological changes in 501Mel cells similar to those observed following siRNA silencing of MITF (Fig 1B). In both cases, cells showed an enlarged, flattened and irregular morphology with extensive cytoplasmic projections. Similar results were observed following infection with lentiviral vectors expressing two different shRNAs directed against BPTF both of which led to diminished BPTF levels whereas MITF expression was unaffected (Fig 1A). ShBPTF silencing also led to strongly diminished SMARCA5 and SMARCA1 protein levels (Fig 1A), although the expression of the corresponding genes was not reduced (see below). As BPTF is so far believed to be exclusive to the NURF complex [20], the loss of SMARCA5 and SMARCA1 suggests that a large fraction of these proteins is associated with BPTF in the NURF complex that is destabilized by BPTF silencing. ShBPTF knockdown therefore leads to a loss of NURF function possibly through its chromatin-remodeling activity.

After 5 days of shBPTF silencing, cells displayed marked morphological changes analogous to those seen following siBPTF silencing and si/shMITF silencing (Fig 1C and 1D). These morphological changes were characteristic of those observed when 501Mel cells enter senescence [12,37] and up to 90% of shBPTF or shMITF silenced cells showed staining for senescence-associated β-galactosidase (Fig 1C and 1D). BPTF silencing therefore induced senescence in 501Mel cells.

Analogous results were observed in several other MITF-expressing melanoma cell lines. ShRNA-mediated BPTF silencing in SK-Mel-28 cells led to reduced cell number and marked morphological changes with many bi-nucleate and multi-nucleate cells (S2A, S2B and S2C Fig). In melanoma MNT1 cells, shBPTF silencing led to a spindle-like bipolar morphology (S2B and S2C Fig), while in 888-Mel cells, BPTF knockdown led to strongly reduced cell numbers with the remaining cells again showing a spindle-like bipolar morphology (S2C Fig). We also investigated BPTF function in MITF-negative 1205Lu cells. In this cell line also, BPTF silencing induced an enlarged, flattened more rounded senescence-like morphology (S2D, S2E and S2F Fig). On the other hand, shMITF silencing had no effect on these cells consistent with the fact that they do not express MITF.

In contrast to the above, shBPTF silencing in a variety of non-melanoma cells such as HeLa (cervical cancer), HEK293T (human embryonic kidney) had no effect on either proliferation or morphology (S3A, S3B and S3C Fig). These observations show that BPTF plays a selective and essential role in melanoma cells that is not seen in non-melanoma cells. Moreover, as BPTF is essential in 1205Lu cells, it may have both MITF-dependent and independent functions in melanoma.

### BPTF and MITF regulate overlapping gene expression programs in 501Mel cells

**Fig 2:**
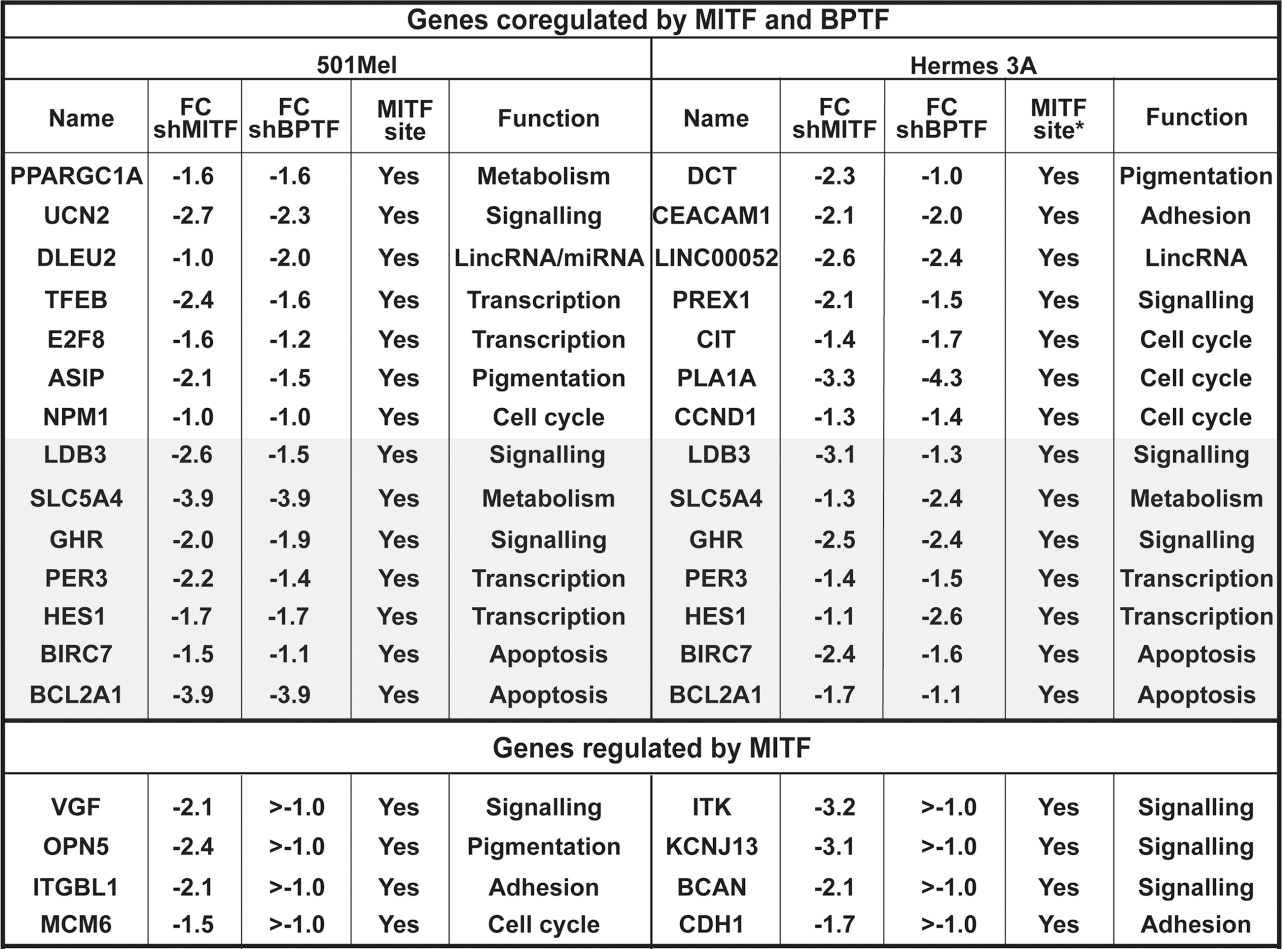
Co-regulated genes in 501Mel and Hermes 3A cells. Examples of genes that are co-regulated by MITF and BPTF in each cell type. The Log2 fold change under each condition is indicated. The shaded area highlights genes that are co-regulated in both cell types. The lower portion shows examples of genes that are regulated by MITF only. All of the genes listed are direct MITF targets with associated MITF-occupied sites in 501Mel cells. The asterisk in the Hermes column indicates that these genes are direct MITF targets in 501Mel cells, but that we have no corresponding ChIP-seq data in Hermes 3A cells.

As BPTF and MITF silencing in 501Mel cells generated similar phenotypes, we performed RNA-seq following shRNA-mediated silencing and compared the de-regulated gene expression programs. Following BPTF knockdown, 494 genes were down-regulated (Fig 1E), enriched in ontology terms associated with regulation of transcription, kinase signalling, pigmentation and cell cycle (Fig 1F and S1 Dataset). 593 genes were down-regulated by MITF knockdown, enriched in cell cycle, in particular in mitosis, consistent with previous observations that MITF silencing leads to severe mitotic defects [12,17]. Comparison of the two data sets identified 191 common repressed genes associated with transcription regulation and kinase signalling (Fig 1E and 1F). Thus, 39% of genes down-regulated by BPTF silencing were also down-regulated by MITF silencing indicating a large overlap between the programs regulated by each factor. To determine the statistical significance of this overlap, we used hypergeometric probability to calculate the representation factor (RF) that determines whether the number of genes in the overlap is higher than expected by chance taking into account the number of genes regulated by MITF and BPTF with respect to the total number of expressed genes. For the common down-regulated genes the RF was 9.3 (p < 6.048e-141) showing the high statistical significance of the overlap. Some examples of co-regulated genes are listed in Fig 2. Moreover, the number of co-regulated genes was highest in the top quartiles of shMITF-regulated genes (44% in quartile 1, S4A Fig). Genes with strongest dependency on MITF were therefore more often co-regulated by BPTF than those of the bottom quartiles whose expression is least affected by MITF loss.

Following BPTF knockdown, 669 genes were up-regulated (Fig 1E and 1F), enriched in cell adhesion, morphology and motion as well as a set of secreted cytokines and growth factors that constitute the senescence associated secreted phenotype (SASP). 748 genes were up-regulated by MITF knockdown, enriched in terms analogous to those of BPTF including an extensive SASP [17,38]. Comparison of the two data sets identified 278 common induced genes, with 41% of genes up-regulated by BPTF silencing also induced by MITF silencing. For the common up-regulated genes the RF was 7.9 (p < 3.495e-191) again showing the statistical significance of this overlap. As noted above for down-regulated genes, the number of co-regulated genes was highest in the top quartiles of shMITF-regulated genes (57% in quartile 1, S4A Fig). Consistent with the similar morphological changes, the common regulated genes were involved in morphology and adhesion as well as the SASP. None of the genes repressed by MITF knockdown were activated by BPTF knockdown and only 8 genes repressed by BPTF knockdown, showed an opposite regulation, being activated by MITF knockdown.

These data show a large and significant overlap between the gene expression programs controlled by BPTF and MITF. Together these two factors positively regulate genes required for proliferation and negatively regulate genes involved in modulating cell morphology and motility.

We determined which up and down-regulated genes are associated with MITF-occupied sites. As described [17], MITF occupies >16000 sites in 501Mel cells. Using a window of +/-30kb with respect to the TSS, taking into account potential regulation by MITF from distant enhancers, identified up to 5694 potential targets (S4B Fig). Of these 176 genes are down-regulated upon MITF silencing consistent with them being directly activated by MITF. Several cell cycle regulators such as *CCND1*, *ANLN* and *CIT* were associated with multiple MITF binding sites (S2 Dataset). Up to 56 genes associated with MITF binding sites were co-regulated by BPTF, including *BIRC7 SHB*, and *BCL2A1* critical regulators of proliferation, apoptosis and survival, *NPM1* an important cell cycle regulator involved in chromosome congression, spindle and kinetochore-microtubule formation required for normal centrosome function [39,40] and *PPARGC1A* (PGC1α) implicated in resistance to oxidative stress and mitochondrial function in melanoma [41,42] (S4C Fig). MITF and BPTF therefore co-regulate these critical MITF target genes. Similarly, up to 187 genes were potentially directly repressed by MITF including several SASP components such as *SERPINE1*, *IL8*, *IL24* and *PDGFB* [see also [17]]. Of these 67 were co-regulated by BPTF such as SASP components *IL8* and *IL24*. BPTF and MITF therefore appear to co-regulate gene expression in a positive and negative manner.

### BPTF and MITF regulate proliferation of Hermes 3A melanocytes

**Fig 3:**
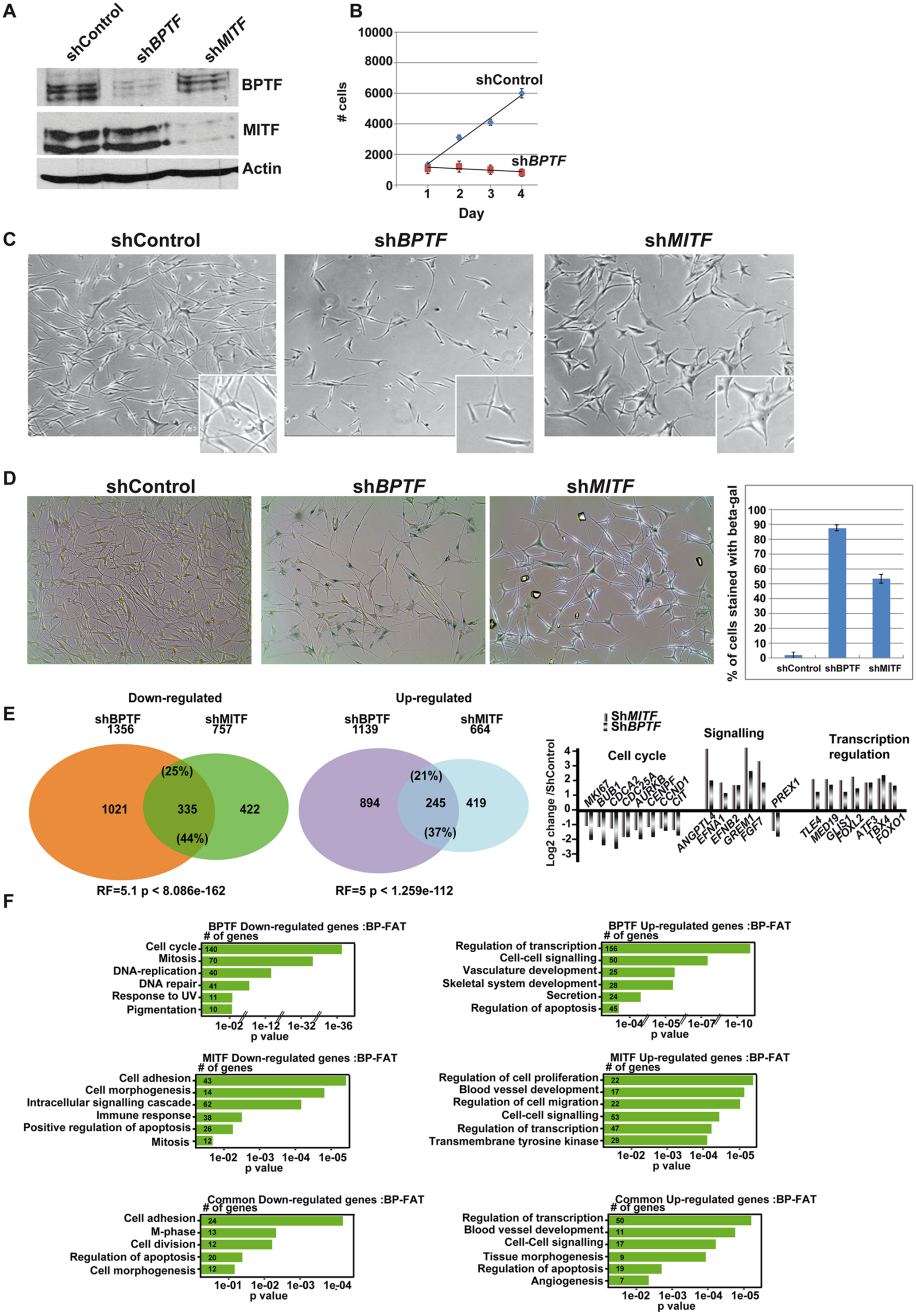
BPTF and MITF are required for the proliferation of Hermes-3A cells. **A.** Western blot showing knockdown of BPTF and MITF in Hermes-3A cells. **B.** Numbers of Hermes-3A cells measured using a MTT cell proliferation kit following shBPTF knockdown. **C**. Phase contrast microscopy of Hermes-3A cells following shBPTF and shMITF knockdown. Magnification X20. Inlays show enlargements. **D**. Staining and quantification of Hermes-3A cells following shBPTF and shMITF knockdown for senescence-associated β-galactosidase. **E.** Venn diagrams illustrate the overlap between up and down-regulated genes following shBPTF and shMITF knockdown. RF shows the representation factor and p value for the overlaps between the up and down-regulated genes sets. Several examples of commonly regulated up and down-regulated genes are indicated based on the results of RNA-seq. **F.** Ontology of genes up and down-regulated by shBPTF and shMITF knockdown and of commonly regulated genes in Hermes 3A cells.

We next investigated the role of BPTF in untransformed human melanocytes by shBPTF silencing in the Hermes 3A cell line. As previously shown, MITF silencing in these cells led to proliferation arrest, morphological changes and entry into senescence [[17] and Fig 3A and 3C]. Fewer cells were also detected following BPTF knockdown, but the cells had a more bipolar morphology compared to the expanded and flattened morphology of the shMITF cells (Fig 3A, 3B and 3C). Almost 85% of shBPTF cells showed senescence-associated **β**-galactosidase staining (Fig 3D). RNA-seq showed that BPTF silencing repressed 1356 genes and up-regulated 1139 genes (Fig 3E and S3 Dataset). The effects of BPTF loss on gene expression were therefore more extensive in these cells than in 501Mel. Down-regulated genes were strongly enriched in cell cycle, mitosis and pigmentation functions (Fig 3F). BPTF is therefore a major regulator of genes required for proliferation of Hermes 3A cells. The up-regulated genes on the other hand were involved in transcription regulation, cell-cell signalling, including many secreted and membrane associated proteins.

The gene expression programs regulated by BPTF and MITF overlapped significantly (RF = 5.1, p < 8.086e-162) as overall 44% of genes down-regulated by shMITF knockdown were also down-regulated by shBPTF (Fig 3E). As seen with 501Mel cells, the genes most strongly regulated by MITF were most often co-regulated by BPTF (62% in the first quartile, S4A Fig). Co-regulated genes were associated with cell cycle/mitosis, cell adhesion and apoptosis (Figs 3F and 2). For example, several critical regulators of cell cycle and mitosis such as *AURKB*, *CDCA2* and *CCND1*, genes associated with MITF occupied sites in 501Mel cells, were repressed under both conditions (Fig 2). Similarly, 37% (RF = 5, p < 1.259e-112) of genes up-regulated by shMITF were also induced by shBPTF (Fig 3E and 3F) including a plethora of signalling molecules and transcriptional regulators.

We next compared the gene expression programs regulated by silencing of BPTF, MITF and BRG1 in Hermes 3A cells. BPTF and BRG1 silencing commonly down-regulated 277 genes. Of these 122 were also regulated upon MITF silencing (S4D Fig). In all, 58% of MITF-regulated genes were co-regulated by either BRG1 or BPTF and 16% regulated by both. BPTF and BRG1 silencing commonly up-regulated 208 genes of which 126 were also up-regulated by MITF silencing (S4D Fig). Overall, 56% of genes up-regulated by MITF were co-regulated by either BRG1 or BPTF and 19% regulated by both. A large fraction of MITF regulated genes are therefore co-regulated by these remodellers in Hermes 3A cells. A similar comparison could not be performed in 501Mel cells were BRG1 regulated a very large number of genes.

The transcriptional programs regulated by MITF in 501Mel and Hermes3A cells were somewhat different as only 172 genes (22%) were down-regulated and only 130 genes (19%) up-regulated in both lines (S4E Fig). Nevertheless, the common down-regulated genes comprised regulators of cell cycle and mitosis defining a critical set of MITF-regulated cell cycle genes and up-regulated genes were principally involved in signalling and cell motion/morphogenesis. An analogous comparison of BPTF regulated genes showed 46% of common down-regulated genes and 34% of up-regulated genes. Common down-regulated genes comprised regulators of cell cycle and up-regulated genes comprised genes involved in signalling and cell motion/morphogenesis (S4F Fig). A small number of genes were also co-regulated by MITF and BPTF in both cells lines (shaded area in Fig 2).

### Bptf acts during melanoblast proliferation and migration

The essential role of BPTF in melanoma and melanocyte cells *in vitro* prompted us to investigate its role in the murine melanocyte lineage *in vivo*. Interrogation of transcriptome data from purified melanoblasts (GFP+ cells, see [43]) and the GFP- cells (mainly keratinocytes) from E15.5 mouse embryos indicated that *Bptf* and *Smarca5* were expressed in both cell types to levels comparable to those of the *Smarca4* (Brg1) and *Pbrm1* subunits of the PBAF complex that is essential for melanoblast development [44], (S1C Fig). However, no significant Smarca1 expression was seen at this stage.

We crossed mice with a floxed *Bptf* gene (*Bptf* ^lox/lox^) with those expressing Cre recombinase under the control of the Tyrosinase enhancer that allows selective inactivation in the developing melanocyte lineage at E9.5-E10.5 [45]. The resulting *Tyr*-Cre/°::*Bptf* ^lox/lox^ mice were further crossed with animals expressing the LacZ reporter gene under the control of the *Dct* promoter that marks cells of the melanocyte lineage (*Tyr*-Cre/°::*Bptf* ^lox/lox^::*Dct*-LacZ/°).

**Fig 4:**
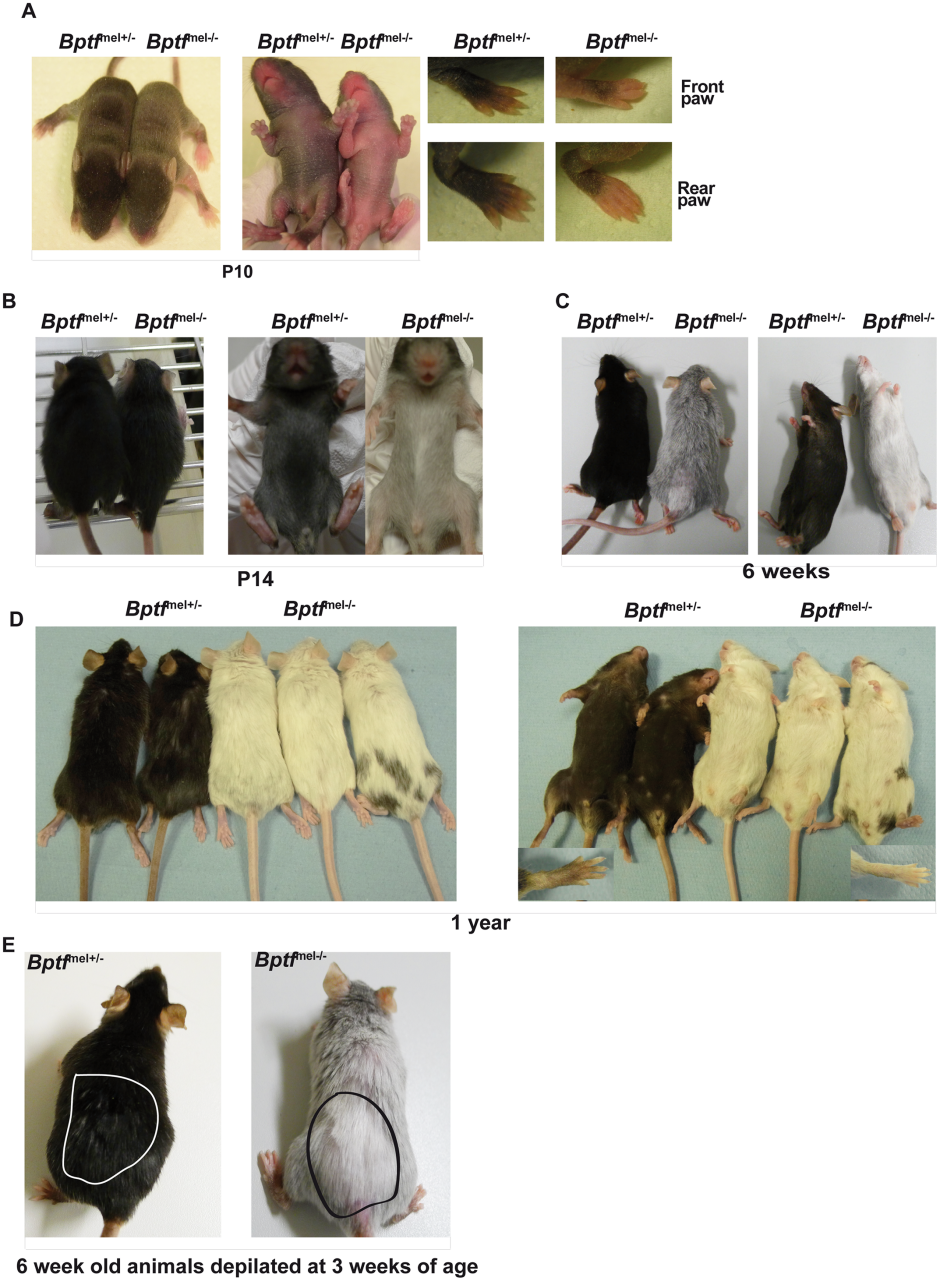
Premature greying of mice lacking Bptf in the melanocyte lineage. **A** Photographs of P10 mice of the indicated genotypes before onset of hair growth. **B-C.** Photographs of 14 day-old and 6 week-old mice of the indicated genotypes to illustrate the characteristics of the first coat and the premature greying phenotype. **D**. Photographs of 1 year-old mice of the indicated genotypes. **E**. Photographs of 6 week-old mice that had undergone depilation at 3 weeks of age. The depilated areas are outlined.

Following crosses of *Tyr*-Cre/°::*Bptf* ^lox/+^ mice, *Tyr*-Cre/°::*Bptf* ^lox/lox^ animals in which Bptf was inactivated in the melanocyte lineage (*Bptf* ^mel-/-^) displayed a pigmentation phenotype characterised by a grey belly, and grey extremities of the paws, ears and tail compared to wild-type mice and heterozygous *Tyr*-Cre/°::*Bptf* ^lox/+^ (*Bptf* ^mel+/-^) littermates (Fig 4A and S5A, S5B and S5C Fig). This phenotype was confirmed upon growth of the first hair at P14 that was grey on the belly with a small and variable white belly spot (Fig 4B and S5B Fig). Otherwise, the dorsal coat was almost indistinguishable from wild-type. Melanoblasts lacking Bptf are therefore viable, but BPTF regulates their proliferation and/or migration.

To better characterise this phenotype, we used the *Tyr*-Cre/°::*Bptf* ^lox/lox^::*Dct*-LacZ/° mice to monitor melanoblast development. The number of *Dct*-LacZ positive melanoblasts was counted at E15.5 on 4 mice of the *Bptf* ^mel-/-^ and *Bptf* ^mel+/-^ genotypes. The migration front on the belly and the paws was similar in both genotypes (S6A and S6B Fig). In contrast, the number of melanoblasts was reduced by around 10% in the *Bptf* ^mel-/-^ foetuses (S6C Fig).

**Fig 5:**
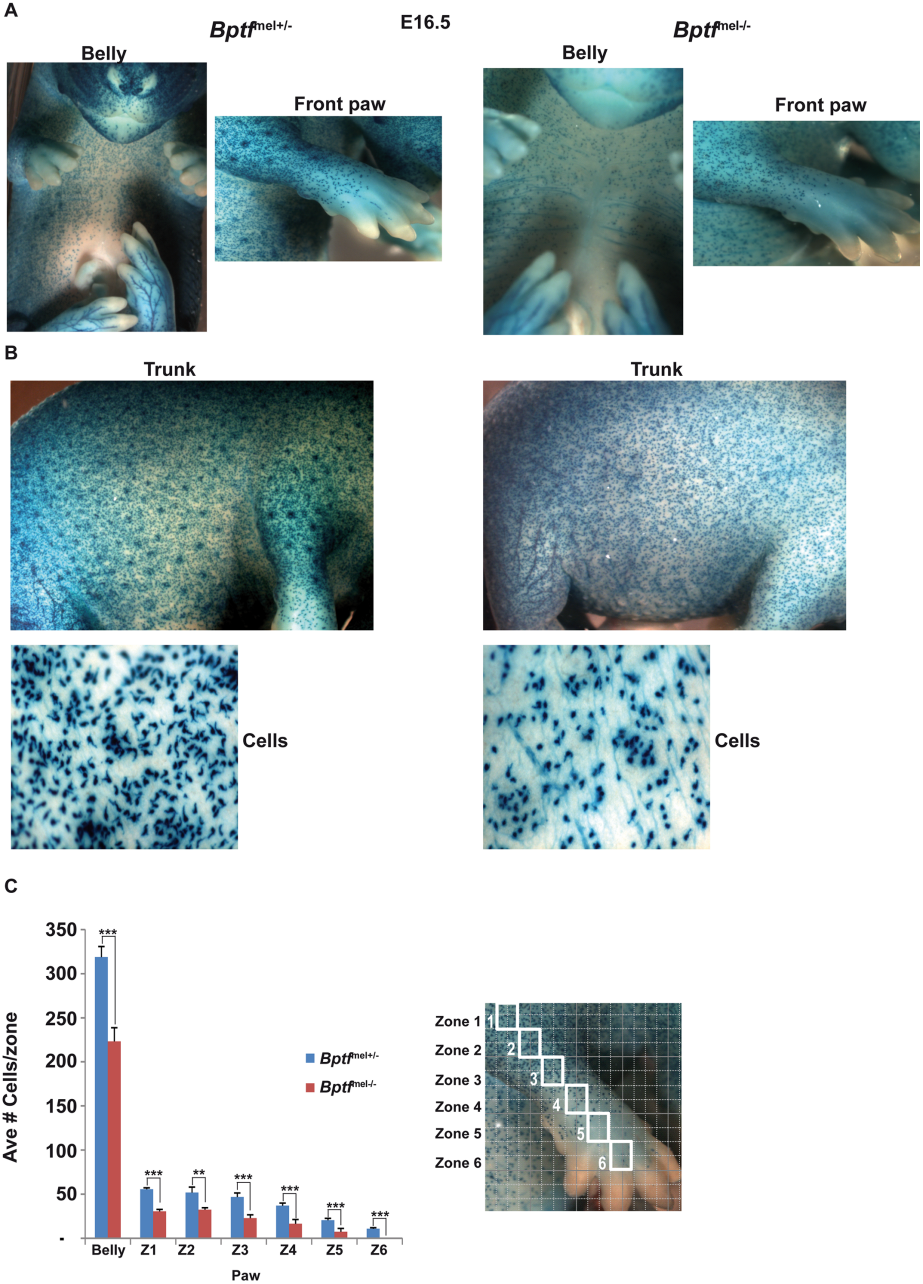
Diminished melanoblast proliferation in Bptf-mutant mice. **A-B.** Photographs of representative *Bptf* ^mel+/-^ and *Bptf* ^mel-/-^ E16.5 foetuses in the *Dct*-LacZ background to identify melanoblasts. Panel A shows the ventral portion and front paw, and panel B the trunk and a zoom of representative cells from the trunk regions. **C.** Quantification of LacZ-labelled melanoblasts in the indicated regions. An example of the grid used is shown over the limb and paw that is divided into zones (Z). Statistical significance of the difference in cell counts between the Bptf^lox/+^ and Bptf^lox/lox^ embryos was assessed using two-tailed, unpaired Student’s *t*-test (**P < 0.01; ***P < 0.001). N = 4.

By E16.5, clear differences in the ventral and limb migration fronts were observed (Fig 5A). In *Bptf* ^mel+/-^, clusters of melanocytes were clearly visible on the trunk that correspond to melanocytes colonising the nascent hair follicles [46] (Fig 5B). Although such clusters were less prominent in the *Bptf* ^lox/lox^ animals, the number of melanoblasts was now reduced by around 20% in the *Bptf* ^mel-/-^ foetuses (Fig 5C), perhaps accounting for their diminished prominence. Futhermore, Bptf also regulated melanoblast morphology as those lacking Bptf were less dendritic and much more rounded. Bptf therefore acts during embryogenesis to regulate melanoblast proliferation, migration and morphology.

Pigmentation of the first coat is provided by the embryonic derived melanoblasts that colonise and terminally differentiate in the hair follicles [6]. As the number of melanoblasts is lower in the ventral region than in the dorsal region even in wild-type mice, the further reduction in melanoblast numbers in the *Bptf* ^mel-/-^ animals during the course of development could partially account for the observed greyer phenotype of the first ventral hair coat. As BPTF regulates expression of the melanin synthesis enzymes in human melanocytes *in vitro* (see above), it is also possible that a reduction in melanin production by the mutant melanocytes would also contribute to the greyer ventral phenotype.

### Bptf is essential for differentiation of melanocytes from adult stem cells

As the *Bptf* ^mel-/-^ animals grew older, their pelage showed progressive greying such that by 3–6 weeks both the ventral and dorsal coat became grey and then finally white (Fig 4C and S5D Fig). The animals maintained this completely white pelage throughout their lifespan (Fig 4D). This premature greying phenotype was fully penetrant and most animals became completely white indicating complete recombination of the *Bptf* alleles during embryogenesis, although some animals showed spotting (for example, animal on the right of Fig 4D) by rare melanocytes derived from melanoblasts that escaped recombination. Recombination of the *Bptf* allele was confirmed by PCR-based genotyping on both neonatal mouse-tail DNA and purified E15.5 melanoblasts (S5E Fig).

Greying was accelerated by depilation of 3-week animals, following which the newly grown hair was white (Fig 4E and S5F Fig). These observations revealed that *Bptf* ^mel-/-^ animals were unable to pigment the pelage from the second post-natal anagen, growth phase of the hair cycle when the new hair follicle is generated, that requires the generation of mature melanin producing melanocytes from the post-natal MSC population. Bptf is therefore required for establishment, maintenance and/or functionality of the MSC population or its derivatives.

**Fig 6:**
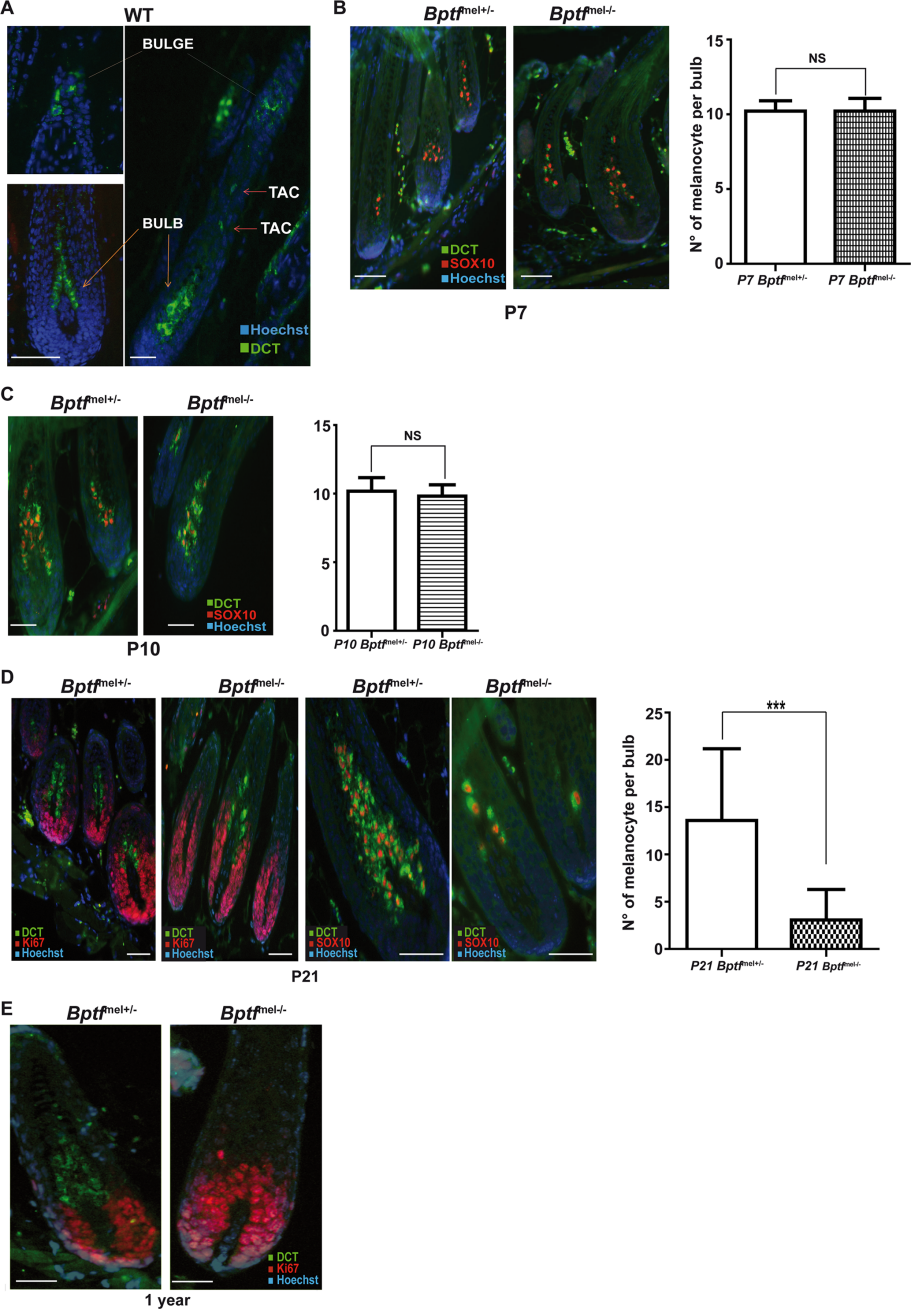
Loss of differentiated melanocytes in the bulb region of post-natal *Bptf* ^mel-/-^ mice. A. Staining of a dorsal wild-type hair follicle with antibody against Dct showing the presence of Dct-labelled cells in the bulge corresponding to MSCs, in the shaft corresponding to TACs and in the bulb corresponding to differentiated melanocytes. B-C. Staining of dorsal hair follicles from mice with the indicated genotypes with antibodies against Dct and Sox10 to detect differentiated melanocytes in the bulb. Quantification is shown on the right. N = 30. D. Staining of dorsal hair follicles from mice with the indicated genotypes with antibodies against Dct and Ki67 to detect melanocytes in the bulb of 21 day-old animals. Quantification is shown on the right. N = 50. E. Staining of dorsal hair follicles from one year-old mice with antibodies against Dct and Ki67 illustrating the absence of melanocytes in the bulb of Bptf-mutant animals. Scale bars represent 50μm. NS = Non significant. ***P < 0.001

To investigate this, we performed immunostaining of hair shafts from the *Bptf* ^mel-/-^ and *Bptf* ^mel+/-^ genotypes at different post-natal stages using antibodies against Dct, staining the MSCs, the TACs and mature melanocytes, against Sox10, labelling TACs and mature melanocytes and against the cell cycle marker Ki67 labelling all proliferating cells in the bulb [6,47,48]. Control staining of a section from an adult wild-type mouse indeed confirmed that Dct stained the mature melanocytes in the bulb along with MSCs in the bulge and TACs (Fig 6A). Staining of dorsal hair shafts from P7 and P10 animals revealed equivalent numbers of Dct-Sox10 stained melanocytes in the bulb region (Fig 6B and 6C). However at 3 weeks the number of Dct stained cells in the bulb strongly decreased in the *Bptf* ^lox/lox^ animals and almost half the shafts were devoid of Dct-stained cells (Fig 6D). In 1-year animals, no Dct-stained cells were detected in the bulb (Fig 6E). The loss of mature bulb melanocytes is in accordance with the progressive loss of pigmentation in the mutant animals and the sustained white coat in older animals.

**Fig 7:**
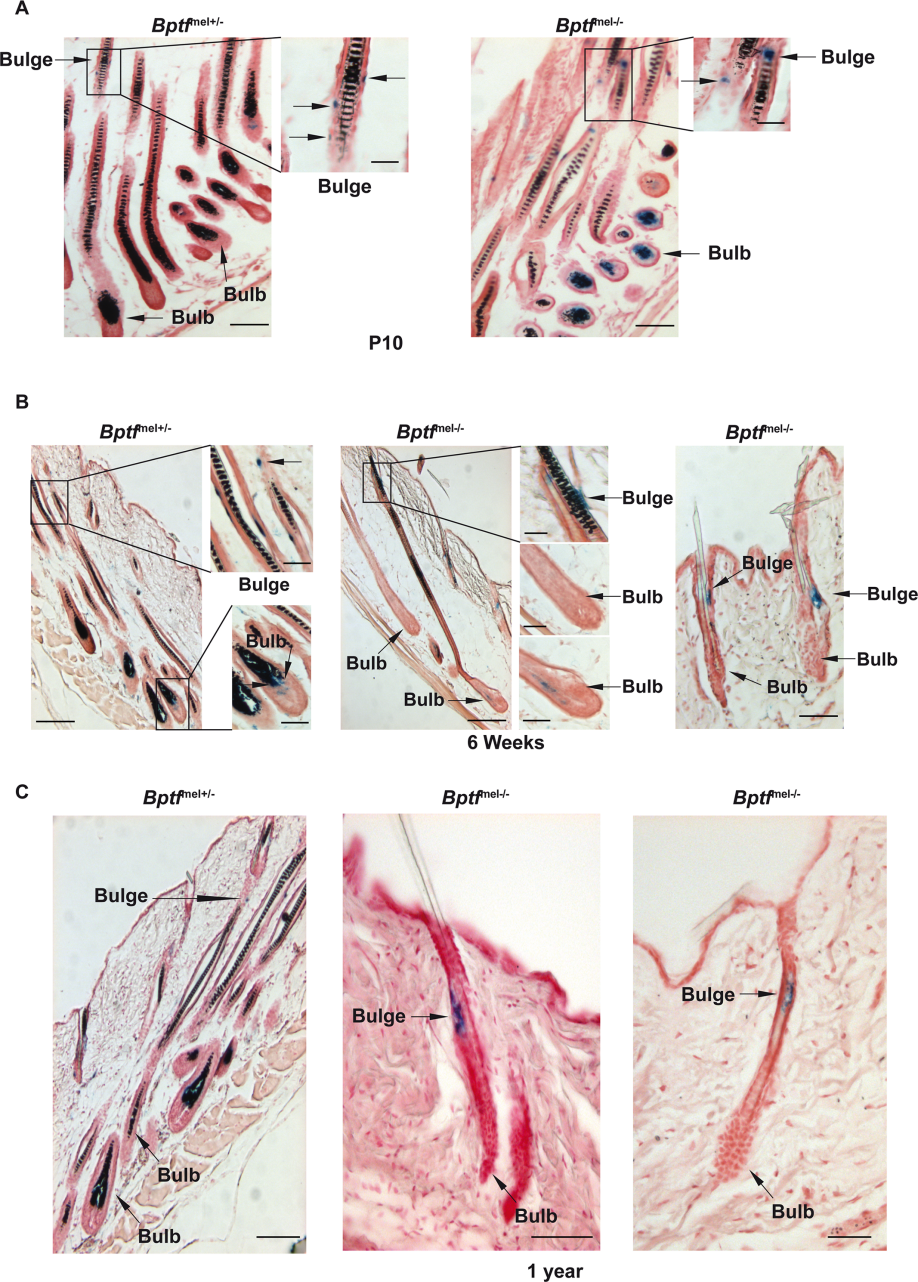
Persistence of a MSC population in adult Bptf-mutant animals. **A**. Staining for *Dct*-LacZ-labelled melanocytes in dorsal hair follicles of P10 mice of the indicated genotypes. Arrows indicate representative examples of MSCs in the bulge region and differentiated melanocytes in the bulb. **B-C** Staining for *Dct*-LacZ-labelled melanocytes in dorsal hair follicles of 6 week-old and one year-old mice. Arrows are as in panel A. Scales bars represent: 20μm in right of panel B and panel C, 25μm in inlays of panels A and B and 50μm in panel A and left and center images of panel B.

In previous mouse models, premature greying was associated with a loss of the MSC population [49–51]. To determine the fate of the MSC population upon Bptf inactivation, we stained sections from the epidermis of the *Tyr*-Cre/°::*Bptf* ^lox/lox^::*Dct*-LacZ/° and *Tyr*-Cre/°::*Bptf* ^lox/+^::*Dct*-LacZ/° animals at different ages for the presence of LacZ to visualize melanocytes. At P10, DCT-LacZ stained melanocytes were seen in the bulbs of the *Bptf* ^mel-/-^ and *Bptf* ^mel+/-^ genotypes and the presence of melanin was clearly visible (Fig 7A). *Dct*-LacZ stained cells were also seen in the bulge regions at this stage. By six weeks, strong staining for melanin persisted in the *Bptf* ^mel+/-^ animals along with the presence of *Dct*-LacZ-stained melanocytes in both the bulge and bulb regions (Fig 7B). In the *Bptf* ^mel-/-^ animals, many shafts devoid of melanin were visible and only rare *Dct*-LacZ-stained melanocytes were seen in the bulb. However, *Dct*-LacZ-positive cells were visible in the bulge region, clearly seen in short telogen/early anagen shafts (Fig 7B, right panel). Staining of one year-old animals that were white, devoid of melanin and *Dct*-LacZ-stained bulb melanocytes also revealed *Dct*-LacZ-positive cells in the bulge region. Thus, an adult MSC population is established and maintained in the absence of Bptf, but these cells are unable to give rise to differentiated pigment producing melanocytes.

**Fig 8:**
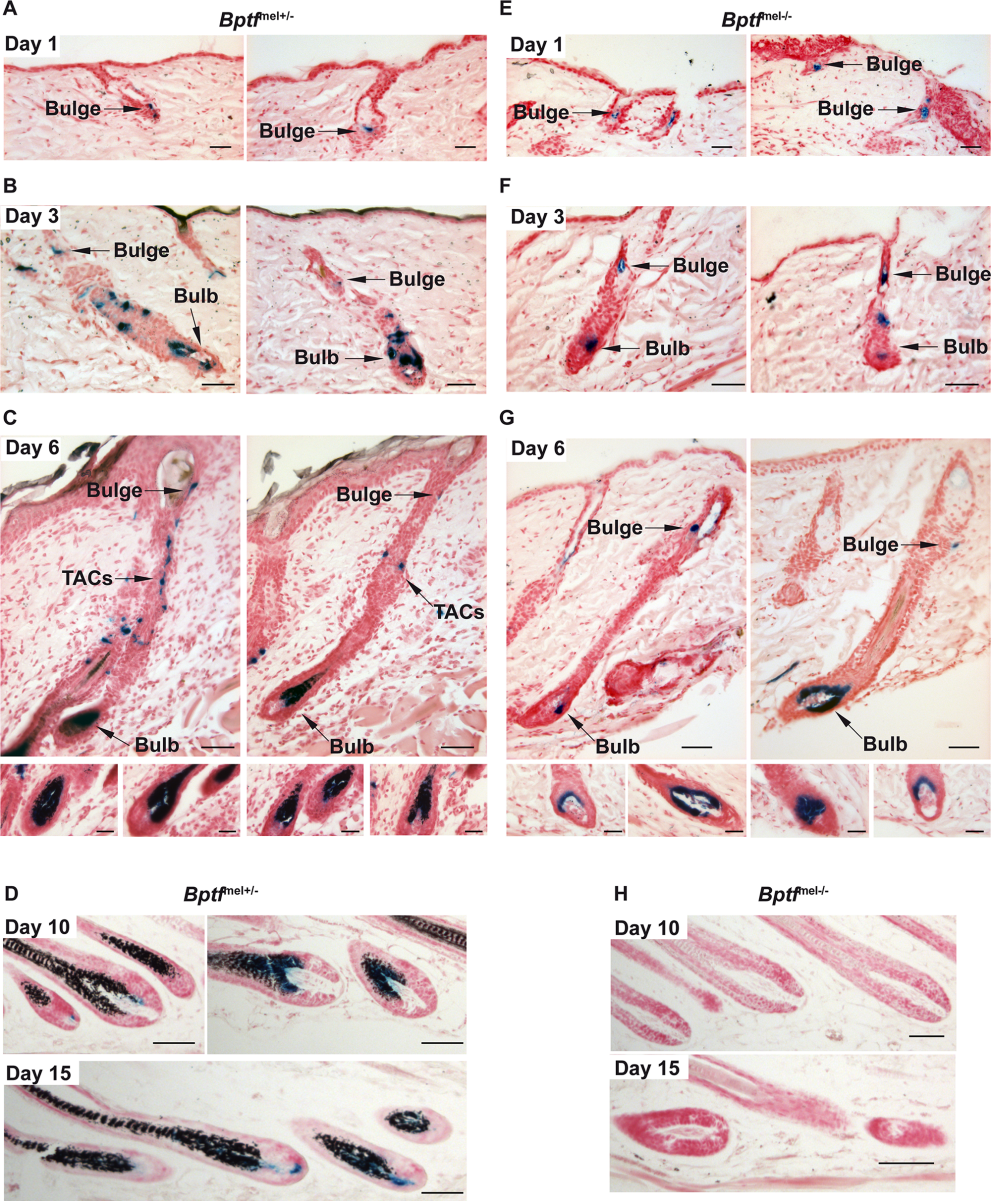
Bptf is required for proliferation and terminal differentiation of adult melanocytes. **A-D.** *Dct*-LacZ staining shows generation of TACs and terminal differentiation of *Bptf* ^mel+/-^ melanocytes 1, 3, 6, 10 and 15 days after depilation. Staining for melanin reveals pigmentation of the newly formed hair. **E-H.** *Dct*-LacZ staining shows reduced number of TACs and lack of differentiated melanin producing *Bptf* ^mel-/-^ melanocytes. The lower panels of C and G show representative examples of bulbs of the two different genotypes. Note the absence of pigmentation of the newly formed hair. All sections were stained for melanin by Fontana Masson and for *Dct*-LacZ-expressing cells. Scales bars represent: 20μm in panels A and E, 25μm in panels B and F and in inlays of panels C and G and 50μm in panels C, D, G, and H.

To ask whether MSCs were able to proliferate and differentiate in response to anagen stimuli, we depilated 4 month-old white animals and monitored the presence of *Dct*-LacZ-stained cells after 1, 3, 6, 10 and 15 days. In *Bptf* ^mel+/-^ animals, short telogen shafts remain in wild-type animals at 1 day following depilation where *Dct*-LacZ stained cells were observed in the bulge region (Fig 8A). By 3 days post-depilation, many TACs could now be seen along with melanocytes that begin to colonise the future bulb (Fig 8B). By 6 days post-depilation, the bulb was filled with mature and pigment-producing melanocytes as seen by staining for *Dct*-LacZ and melanin and numerous TACs could still be observed (Fig 8C). After 10 and 15 days strong *Dct*-LacZ-staining was maintained in the bulb and the newly grown hair was pigmented (Fig 8D).

Immediately after depilation of *Bptf* ^mel-/-^ animals, *Dct*-LacZ stained cells were observed in the bulge region, but after 3 days fewer TACs and bulb melanocytes were visible compared to *Bptf* ^mel+/-^ animals (Fig 8E and 8F). Similarly, at 6 days post-depilation, while *Dct*-LacZ stained cells were visible in the bulge, staining of the bulbs was less prominent and more variable with many bulbs showing only few melanocytes and very few residual TACs were observed (Fig 8G). By 10 and 15 days, *Dct*-LacZ stained cells were no longer seen in the bulb region (Fig 8H). Moreover, no melanin was made from the bulb melanocytes and the out-growing hair was white in accordance with the fact that these animals were white both before and after depilation.

**Fig 9:**
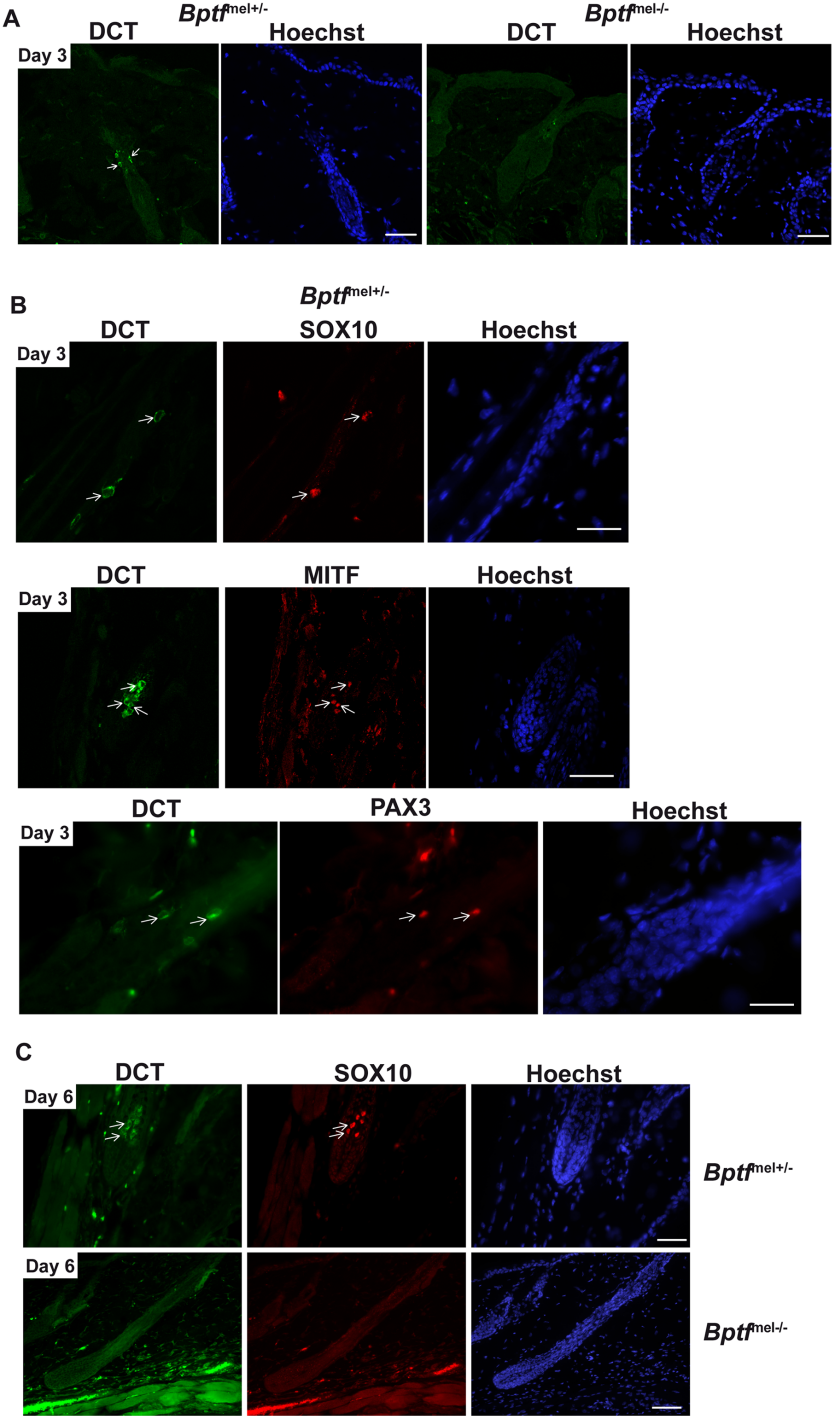
Bptf acts upstream of Mitf in differentiating adult melanocytes. **A.** Confocal microscopy images showing staining for endogenous Dct in *Bptf* ^mel+/-^ and *Bptf* ^mel-/-^ hair follicles 3 days after depilation. **B.** Confocal microscopy images showing staining for endogenous Dct, Mitf, Pax3 and Sox10 in *Bptf* ^mel+/-^ hair follicles. Arrows show double labelled cells. **C.** Staining for endogenous Dct and Sox10 identifies maturing *Bptf* ^mel+/-^ bulb melanocytes 6 days after depilation, but an absence of staining in *Bptf* ^mel-/-^ bulbs. Scale bars represent 50μm.

We also used immunostaining to detect endogenous Dct after depilation. At day 3, Dct labelled cells could be seen in the bulge region of *Bptf* ^mel+/-^ animals (Fig 9A) as well as in migrating TACs and cells colonising the bulb (Fig 9B). Moreover, Dct-labelled cells expressing Pax3, Mitf and Sox10 were also observed at day 3 (Fig 9B and 9C). In contrast, no expression of Dct or of any of the other markers was detected in the *Bptf* ^mel-/-^ animals (Fig 9A). At days 6 and 10, antibody staining showed endogenous Dct in the *Bptf* ^mel+/-^ bulb melanocytes that were also labelled for Sox10 (Fig 9C and S7 Fig). Melanocytes in bulbs from these animals also expressed Pax3, Mitf and the HMB45 melanosome marker (S7C Fig). In contrast, no staining for any of these proteins was seen in the *Bptf* ^mel-/-^ bulbs. Thus, in absence of Bptf, the *Dct*-LacZ-labelled cell population did not express endogenous Dct and their expansion at anagen was not associated with detectable expression of Mitf, Pax3 and Sox10. In addition, the few cells that migrated to the bulb by day 6 also did not express these melanocyte markers. These observations indicate that Bptf acts at an early stage in the generation of differentiated mature melanocytes from the adult MSC population.

**Fig 10:**
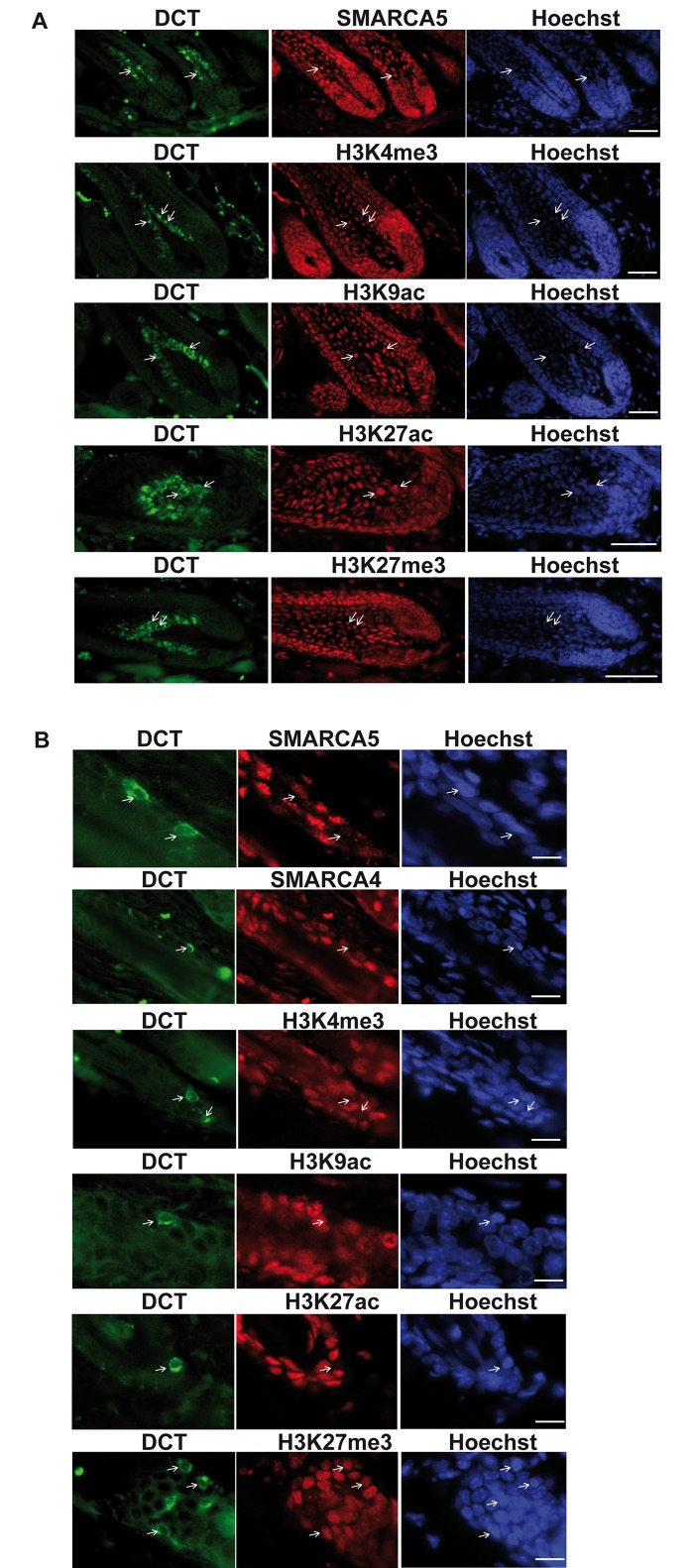
A transcriptionally repressed chromatin state in MSCs. **A**. Staining of hair follicle bulbs with antibodies against Dct and the indicated chromatin remodellers and chromatin modifications. Representative Dct-expressing melanocytes are indicated by arrows. **B.** Staining of the bulge region with antibodies against Dct and the indicated chromatin-remodellers and chromatin modifications. Dct-stained cells with diminished expression of the corresponding marks are indicated by arrows. Scale bars represent 50μm in panel A and 20μm in panel B.

It was previously reported that adult MSCs display a down-regulation of RNA polymerase II (Pol II) transcription witnessed by diminished staining for phosphorylated serine 2 of the C-terminal domain (CTD) of the largest subunit and for Cdk9 [52]. During the course of this study, we tested several antibodies for their ability to detect Bptf in the hair follicle. None of the tested antibodies detected Bptf in immunostaining of hair follicles, whereas a commercial antibody gave a strong nuclear staining for Smarca5 in the keratinocyte population as well as the Dct-expressing bulb melanocytes (Fig 10A). Interestingly however, Dct-expressing MSCs in wild-type mice selectively showed strongly diminished Smarca5 staining suggesting its down-regulation in these transcriptionally silent cells (Fig 10B). A similar result was seen for the Smarca4 (Brg1) chromatin remodeller. To further investigate the transcriptionally repressed state, we stained hair follicles for marks of active chromatin (H3K4me3, H3K9ac and H3K27ac) and the repressive mark H3K27me3. All of these antibodies gave strong nuclear staining in keratinocytes and Dct-expressing bulb melanocytes (Fig 10A). In contrast, Dct-expressing MSCs showed strongly diminished staining for all of the active marks, but not for the repressive H3K27me3 mark (Fig 10B). These data extend the previous observations showing not only diminished phosphorylation of the Pol II CTD and Cdk9, but also reduced expression of the Brg1 and Smarca5 chromatin remodellers and diminished active chromatin marks.

Together these observations support the idea that MSCs enter into a transcriptionally silent state. Emergence from this state to properly reactivate expression of the melanocyte differentiation genes and generate mature melanocytes requires Bptf.

## Discussion

### An essential and specific role for BPTF/NURF in melanoma and melanocyte cells *in vitro*

We show that peptides for the NURF components BPTF, SNF2H, SNF2L and RBBP4 were found in the MITF interactome. As previously described no peptides for these proteins were found in the control FLAG-HA immunoprecipitations from 501Mel cells with native MITF [17]. We did not detect peptides for the smaller BAP18 and HMG2L1 subunits. Whether this is because the small number of peptides from these proteins was missed in the mass-spectrometry or whether they are not part of the complex in 501Mel cells remains to be determined. Mass-spectrometry and immunoblot showed that NURF subunits were detected only in the chromatin-associated fraction indicating that MITF and NURF preferentially interact on chromatin.

Immunoprecipitation showed that endogenous NURF was co-precipitated only in the cells expressing tagged MITF demonstrating the specificity of the interaction with MITF. Lack of ChIP-grade BPTF antibodies has hampered our attempts to identify sites on the genome where MITF and BPTF co-localize. It has previously been shown that BPTF localizes almost uniquely to the active TSS by virtue of the interactions between its PHD and bromodomain with the histone modifications at the TSS [29]. However, these experiments were performed with a truncated epitope tagged protein containing only the PHD and bromodomains and thus it remains possible that BPTF may be recruited to other regions of the genome not by interaction with chromatin marks, but via interactions with transcriptional activators such as MITF as is seen with the PBAF complex [17].

NURF components are expressed in all tested melanoma cell lines and shRNA-mediated BPTF silencing showed its essential role in a variety of MITF-expressing melanoma cell lines, including 501Mel, MNT1, SK-Mel-28, and 888-Mel as well as the MITF-negative 1205Lu cell line where it must act independently of MITF. In contrast, BPTF silencing in a series of non-melanoma cell lines such as HeLa, H293T had no detectable effect, in accordance with previous observations showing *Bptf* ^-/-^ ES cells and MEFs proliferate almost normally *in vitro*, and that thymocytes do not exhibit any proliferation or survival defects *in vivo* [33,53]. There is therefore no general requirement for BPTF for proliferation, but rather a specific requirement in melanoma cells that is both MITF-dependent and independent.

Upon BPTF silencing, 501Mel cells adopted a morphology similar to that seen upon MITF knockdown, developed a SASP and showed senescence-associated β-galactosidase staining. Comparative analyses showed that 39% of shBPTF down-regulated and 41% of up-regulated genes were regulated in an analogous manner by shMITF. Genes such as *BIRC7*, *BCL2A1*, and *NPM1* that have roles in survival and/or in cell cycle regulation were identified as potential direct MITF targets with multiple MITF-occupied sites often close to the transcription start sites, whose expression is co-regulated by BPTF. Similarly, MITF and BPTF co-repress SASP genes like *SERPINE1*, *IL24*, *PDGFB*, and *CYR61* as well as *ZEB1* that has a crucial role in melanoma progression [54,55]. The above observations support the idea that MITF and BPTF positively co-regulate expression of genes involved in proliferation, but co-repress genes controlling cell motility and invasive properties. Nevertheless, BPTF likely acts as a cofactor for other transcription factors in MITF-negative melanoma cells and there are clearly genes regulated by BPTF, but not MITF, in MITF-expressing lines. Acting as a cofactor for MITF is therefore only one facet of BPTF function in melanoma cells.

While this manuscript was in preparation, Dar et al [56] reported the implication of BPTF in human melanoma. In agreement with our results they showed that BPTF silencing in MITF-negative 1205Lu cells arrested their proliferation. More importantly, they showed that BPTF expression is increased in human melanomas, where the corresponding gene is often amplified and that these increases are predictive of poor outcome. While these observations identified BPTF as a prognostic factor for melanoma progression, they did not provide a molecular basis for BPTF function in melanoma. We show here that BPTF acts as a cofactor for MITF in regulating critical cell cycle, invasion, motility and apoptosis genes thus providing a molecular mechanism by which BPTF promotes melanoma growth and progression.

### Bptf is essential for production of differentiated adult melanocytes

Inactivation of Bptf in melanoblasts does not impair their viability. Instead, Bptf regulates melanoblast proliferation and migration with by E16.5, a 20% reduction in the number of melanocytes, an alteration in their morphology and a less advanced migration front. The altered melanoblast morphology with reduced dendriticity may further reflect their impaired migration. Although expression of *RAC1*, an important regulator of melanoblast migration [57] is not affected in shBPTF melanocytes *in vitro*, expression of *RAC2* is down-regulated along with *PREX1* another important regulator of melanoblast migration [58]. Moreover, MITF and BPTF co-regulate *PREX1* expression in melanocytes *in vitro* and the corresponding gene locus comprises multiple MITF-occupied sites [44]. *PREX1* is therefore a direct MITF target gene co-regulated by BPTF in melanocytes *in vitro* and controlling melanoblast migration *in vivo*. While these *in vivo* effects recapitulate to some extent the function of BPTF in regulating melanocyte cell cycle and morphology *in vitro*, Bptf is clearly not essential as a cofactor for Mitf-driven melanoblast development as could have been implied from the observation that Bptf is essential in melanoma/melanocyte cells *in vitro*.

The first cycle of hair growth is pigmented by embryonic melanoblasts that colonize the developing hair follicles and differentiate into mature melanocytes. The almost normal first black dorsal coat of the *Bptf* ^mel-/-^ animals reflects the presence of abundant melanoblasts rendering this region less sensitive to the reduction in melanoblast numbers. The ventral region on the other hand, normally comprises less melanoblasts and hence is more sensitive to the reduced number and migration of melanoblasts in the mutant animals. Nevertheless, by 3–4 weeks as the first coat is discarded and regenerated by the second anagen phase, the mutant animals show progressive greying of both the dorsal and ventral coats. Greying is accentuated by 5–6 weeks when almost all the first coat has been exchanged. Depilation of 3 week-old animals results in the outgrowth of white hair indicating that mutant animals are unable to pigment the hair of the second anagen.

In contrast to the first anagen where pigmentation comes from terminally differentiated embryonic melanoblasts [6], the melanocytes pigmenting the second anagen are derived from post-natal MSCs. The inability of the mutant mice to pigment the hair from the second anagen phase suggests either, the lack of the MSC population, MSCs that are unable to respond to signals that induce their proliferation and/or differentiation at anagen, or a defective proliferation and differentiation of the TACs. The presence of *Dct*-LacZ-positive cells in the bulge region of the hair follicle at P10 and at all subsequent stages including as late as one year when the mice have been completely white for more than nine months shows that Bptf is not required to establish and maintain a MSC population. However, diminished numbers of TACs and bulb melanocytes are observed following depilation-induced anagen of mutant adult animals. These TACs do not express endogenous Dct, Mitf, Sox10 or Pax3 and by 6 days after depilation reduced numbers of *Dct*-LacZ-positive cells are present in the bulb while by 10 days, *Dct*-LacZ expressing cells can no longer be detected. The lower numbers of *Dct*-LacZ-stained TACs and bulb melanocytes may be accounted for by their premature death or by switching off of the *Dct*-LacZ reporter. We cannot exclude the possibility that cells previously stained by *Dct*-LacZ remain in the hair follicle in an undifferentiated state with no expression of melanocyte markers. It is also interesting to note that Dct, and Sox10 staining was also strongly reduced in three week old animals. While it is possible that the negatively stained shafts represent very early second anagen phase shafts, it is more probable that these represent first anagen shafts where there is premature death of the melanocytes or a loss of their melanocyte marker expression. Bptf may therefore be required to maintain the viability and/or the terminally differentiated character of these melanocytes.

These observations show that *Bptf* ^mel-/-^ MSCs are stimulated to proliferate at anagen and fulfill the events necessary to maintain the MSC population throughout the multiple anagens in the life of the animal. Nevertheless, MSCs established just after birth in absence of Bptf, inactivated at an early stage of melanoblast development, are abnormal as they do not express detectable levels of endogenous Dct. This discrepancy with *Dct*-LacZ staining highlights a differential requirement for Bptf for expression of the endogenous *Dct* locus compared to the exogenous transgene reflecting the fact that these two genes are in different chromosomal localizations (chromosome 4 for *Dct*-LacZ and 14 for *Dct*) and chromatin environments and hence show a differential requirement for Bptf/NURF for their expression.

The results reported here together with previous studies show that MSCs undergo a down-regulation of Pol II transcription [52] and display strongly diminished levels of chromatin remodellers and active chromatin marks. All of these observations are consistent with the entry of MSCs into a transcriptionally repressed chromatin state, between the anagen phases. Bptf, presumably via the ATP-dependent chromatin-remodelling activity of NURF, is essential for reactivation of the melanocyte gene expression program at anagen, the subsequent normal proliferation of TACs and their differentiation into mature melanocytes. This is the major defect seen *in vivo*, it is a unique and complex physiological situation that cannot be easily mimicked by cell lines *in vitro*. A specific requirement for Bptf upon reactivation of the MSCs would also explain why Bptf is not essential for differentiation of embryonic melanoblasts that have not undergone a prolonged period of stem cell associated quiescence. It is also noteworthy that the BPTF and SMARCA5 subunits of NURF were identified in an siRNA screen as factors required to maintain keratinocyte stem cells in an undifferentiated state *in vitro* [35]. BPTF is not required to maintain MSCs in an undifferentiated state *in vivo*, but rather is required for their differentiation. BPTF may therefore play opposing roles in keratinocyte and melanocyte stem cells.

Wnt signaling is believed to play an important role in reactivation of MSCs at anagen, [59]. Deletion of Ctnnb1 in melanocytes results in a loss of differentiated progeny and hair greying, but the MSC population is maintained, a phenotype similar to that observed here. Bptf may therefore act downstream of Wnt prior to Mitf induction an observation reminiscent of the situation in Drosophila where NURF is required for Wnt signaling [60].

The role of BPTF/NURF in melanocytes differs from that of BRG1/PBAF that also interacts with MITF [17]. While BRG1 and BPTF are essential in melanocytes and melanoma cells *in vitro*, they regulate overlapping but distinct gene expression programs. Furthermore, mice lacking Brg1 in melanocytes are born with a complete absence of pigmentation and no identifiable melanocytes in the hair follicles showing an essential role for Brg1 in melanoblast development, whereas Bptf is essential only for reactivation of the MSC population. Our results therefore define specific and distinct roles for the PBAF and NURF chromatin remodelling complexes in epigenetic regulation of gene expression in melanocytes and melanoma.

The phenotype observed here is unique and distinct from previous mouse mutants where premature greying was ascribed to a progressive loss of the stem cell population [61]. For example, targeted ablation of Bcl2 in the melanocyte lineage results in loss of the MSC population showing that it is required for their survival [51]. Similarly, melanocyte-specific knockout of Notch signalling components shows the requirement of this pathway to maintain the MSC population [49,62,63]. Bptf knockout on the other hand does not deplete the MSC population that persists throughout life, but plays an essential role in their differentiation.

## Materials and Methods

### Immunopurification and western blot

Cell extracts were prepared essentially as previously described and subjected to tandem Flag-HA immunoprecipitation [17,64]. MITF was detected by antibody ab-1 (C5) from Neomarkers, BPTF by a rabbit polyclonal antibody generated and kindly donated by Dr. J. Landry as described [33], and SMARCA1 and SMARCA5 were detected using antibodies generated and kindly donated by Dr P. Becker as described [65].

### Mice

Mice were kept in accordance with the institutional guidelines regarding the care and use of laboratory animals and in accordance with National Animal Care Guidelines (European Commission directive 86/609/CEE; French decree no.87–848). All procedures were approved by the French national ethics committee. Mice with the following genotypes have been described elsewhere: conditional Bptf^lox/lox^ [33], *Tyr*-Cre [45] and *Dct*-LacZ [66] Genotyping of F1 offspring was carried out by PCR analysis of genomic tail DNA with primers detailed in the respective publications.

### LacZ-staining of embryos and epidermis

E15.5 and E16.5 embryos were washed in PBS and fixed in 0.25% gluteraldehyde in PBS for 45 min at +4°C, after which they were washed with PBS for 15 min at +4°C. Embryos were incubated with permeabilization solution (100 mM phosphate buffer pH 7.4, 2mM MgCl2, 0.01% sodium deoxycholate, 0.02% NP40) for 30–45 min at room temperature (RT). Staining was performed overnight at 37°C with permeabilization buffer containing 5mM potassium ferricyanide and potassium ferrocyanide (Sigma) and 0.04% X-gal solution (Euromedex). The samples were post-fixed for 3 h in 4% paraformaldehyde at RT and washed in PBS overnight at +4°C. To count embryonic melanoblasts, photos were taken with a Nikon AZ100 Multizoom microscope (Nikon, Tokyo, Japan) and defined regions were analyzed with Photoshop grid counter.

For epidermal samples, skin biopsies at the indicated stages were isolated, cut into small pieces (4mm x 2mm) and treated with the same protocol as the embryos, with the exceptions of overnight staining at RT and an overnight post-fixation in 4% paraformaldehyde. For further immunohistochemical analysis, samples were dehydrated, embedded in paraffin and sectioned at 10 μm. Sections were subsequently stained with nuclear fast red (Abcam) and, when indicated, the Fontana Masson kit (Abcam) and pictures were taken with a brightfield microscope.

### Immunostaining

Biopsies of dorsal skin were isolated, cut into small pieces, fixed overnight in 4% paraformaldehyde, washed with PBS, dehydrated, paraffin imbedded and sectioned at 5 μm. For antigen retrieval, the sections were incubated with 10mM sodium citrate buffer, within a closed plastic container placed in a boiling waterbath, for 20 min. Sections were permeabilised with 3x5 min 0.1% Triton in PBS, blocked for 1h in 5% skin milk in PBS, and incubated overnight in 5% skin milk with primary antibodies. The following antibodies were used: goat anti-Dct at dilution of 1/1000 (Santa Cruz Biotechnology, sc-10451), rabbit anti-Ki67, at 1/500 (Novocastra Laboratories, NCL-Ki67p), rabbit anti-Sox10, at 1/1000 (Abcam, ab155279), H3K4me3 (04–745 Millipore), H3K9ac (07–352 Millipore), H3K27me3 (CS200603 Millipore), H3K27ac (39133 Active motif) SMARCA5 (Abcam ab72499). Sections were washed 3x5 min 0.1% Triton in PBS, and incubated with secondary antibodies, Alexa 488 donkey-anti-goat, and Alexa 555 donkey-anti-rabbit (Invitrogen) for 1 h. Sections were subsequently incubated with 1/2000 Hoechst nuclear stain for 10 min. Sections were washed 3x5 min in PBS, dried, mounted with Vectashild, and coverslip immobilized with nail polish.

### Purification of melanoblasts

Melanoblasts were isolated according to a protocol adapted from Van Beuren and Scambler [67]. Briefly, the trunk epidermis of E14.5-E15.5 *Dct-*LacZ::*Tyr*-Cre::*Bptf* ^lox/+^ embryos was dissociated into a single cell suspension and LacZ-positive cells were labelled using the DetectaGene Green CMFDG LacZ Gene Expression kit (Molecular Probes) and isolated by FACS prior to genotyping.

### Cell culture, and shRNA silencing

Melanoma cell lines SK-Mel-28, 501Mel, MNT1, 888Mel and 1205Lu were grown in RPMI 1640 medium (Sigma, St Louis, MO, USA) supplemented with 10% foetal calf serum (FCS). HEK293T, HeLa and fibroblast cell lines were grown in Dulbecco’s modified Eagle’s medium supplemented with 10% FCS and penicillin/streptomycin (7.5 μg/ml). Hermes-3A cells were grown in RPMI 1640 medium (Sigma) supplemented with 10% FCS, 200nM TPA, 200pM cholera toxin, 10ng/ml human stem cell factor (Invitrogen), 10 nM endothelin-1 (Bachem) and penicillin/streptomycin (7.5 μg/ml). Hermes 3A cells were obtained from the University of London St Georges repository.

All lentiviral shRNA vectors were obtained from Sigma (Mission sh-RNA series) in the PLK0 vector and virus was produced in HEK293T cells according to the manufacturers protocol. Cells were infected with the viral stocks and after 5 days (or as indicated in the Figure legends) of puromycin selection (3 μg/ml), cells were photographed and collected for preparation of cell lysates or isolation of RNA. In each case between 5X10^5^-1X10^6^ cells were infected with the indicated shRNA lentivirus vectors and all experiments were performed at least in triplicate. The following constructs were used shBPTF (TRCN0000319152, TRCN0000319153), shMITF (TRCN0000019119). For lentivirus infection, virus was produced in 293T cells according to the manufacturers protocol described. The siRNA knockdown of BPFT was performed with ON-TARGET-plus human SMARpool (L-010431-00) purchased from Dharmacon Inc. (Chicago, Il., USA). Control siRNA directed against luciferase was obtained from Eurogentec (Seraing, Belgium). siRNAs were transfected using Lipofectamine RNAiMax (Invitrogen, La Jolla, CA, USA).

### Senescence-associated β-galactosidase assay

The senescence-associated β-galactosidase staining kit from Cell Signaling Technology (Beverly, MA, USA) was used according to the manufacturer’s instructions to histochemically detect β-galactosidase activity at pH 6.

### mRNA preparation, quantitative PCR and RNA-seq

mRNA isolation was performed according to standard procedure (Qiagen kit). qRT-PCR was carried out with SYBR Green I (Qiagen) and Multiscribe Reverse Transcriptase (Invitrogen) and monitored using a LightCycler 480 (Roche). Actin gene expression was used to normalize the results. Primer sequences for each cDNA were designed using Primer3 Software and are available upon request. RNA-seq was performed essentially as previously described [68]. Gene ontology analyses were performed using the functional annotation clustering function of DAVID (http://david.abcc.ncifcrf.gov/).

## Supporting Information

S1 Dataset Excel spread sheet of genes specifically and commonly regulated by BPTF and MITF knockdown in 501Mel cells along with the gene ontology for each gene set. (XLSX)

S2 Dataset Excel spread sheets show genes associated with an MITF-occupied site in 501Mel cells within 30kb of the corresponding TSS and that are either up or down-regulated by MITF and BPTF silencing. The gene ontology for each gene set is also shown.
(XLSX)

S3 Dataset Excel spread sheet of genes specifically and commonly regulated by BPTF and MITF knockdown in Hermes 3A melanocytes cells along with the gene ontology for each gene set. (XLSX)

S1 Fig NURF associates with MITF. **A.** The immunoprecipitated material from the soluble nuclear extract (SNE) and chromatin-associated extract (CAE) was analysed by mass-spectrometry. The peptides identified for BPTF, SMARCA1 and SMARCA5 in the chromatin-associated fraction are listed according to their MH+ score. **B** Immunoblot detection of MITF, BPTF, SMARCA1 and SMARCA5 in FLAG-HA immunoprecipitations of the indicated extracts (CE is cytoplasmic extract) from cells expressing FLAG-HA tagged or native MITF. **C.** Expression of SMARCA1, SMARCA5 and BPTF in a panel of melanoma cells lines grown *in vitro* (upper panel) and in developing melanoblasts and keratinocytes (lower panel). **D.** Total cell extracts were prepared from the indicated cell lines and the presence of the NURF proteins detected by immunoblotting. Note that BPTF is a 400 kDa protein that is extremely sensitive to proteolysis explaining the presence of multiple species.
(TIF)

S2 Fig BPTF is essential in melanoma cells. **A.** Western blot showing knockdown of BPTF and MITF in SK-Mel-28 cells. **B**. Cell numbers for SK-Mel-28 and MNT1 cells following BPTF knockdown. **C.** Phase contrast microscopy of SK-Mel-28, MNT1 and 888Mel cells following BPTF knockdown. Magnification X20. **D.** Western blot showing knockdown of BPTF and absence of MITF in 1205Lu cells. **E.** Arrested growth of 1205Lu melanoma cells following BPTF knockdown. **F**. Phase contrast microscopy of 1205Lu cells following BPTF and MITF knockdown. Magnification X20.
(TIF)

S3 Fig Effect of BPTF silencing in non-melanoma cells. **A.** Western blot showing knockdown of BPTF in HeLa and HEK293T cells. **B**. Proliferation of HeLa and HEK293T cells is unaffected by BPTF knockdown. **C.** Morphology of HeLa and HEK293T cells is unaffected by BPTF knockdown. Magnification X20.
(TIF)

S4 Fig MITF and BPTF regulated gene expression programs. **A**. The genes regulated by MITF in 501Mel and Hermes 3A cells are divided in quartiles based on their fold change after shMITF silencing. The % of MITF-regulated genes in each quartile co-regulated by BPTF is represented. **B**. Venn diagrams illustrate the overlap between up and down-regulated genes following shBPTF and shMITF knockdown in 501Mel cells and genes showing an associated MITF-occupied site in ChIP-seq experiments in a +/-30 kb window with respect to the TSS. **C.** UCSC screenshots of the *BIRC7*, *NPM1*, *SHB*, *PPARGC1A* and *BCL2A1* genes that are associated with MITF-occupied sites and are down-regulated by MITF and BPTF silencing. HA-MITF shows the ChIP-seq track for HA-tagged MITF and arrows indicate representative MITF-occupied sites. HFM indicates the human foreskin melanocyte H3K27ac ChIP-seq track showing promoter and enhancer elements active in the melanocyte lineage. **D.** Venn diagrams illustrate the overlap between genes up and down-regulated by shBPTF, shMITF and shBRG1 in Hermes 3A cells. **E-F** Venn diagrams illustrate the overlap between genes up and down-regulated by shBPTF and shMITF in 501Mel and Hermes 3A cells. Several examples of commonly regulated up and down-regulated genes are indicated.
(TIF)

S5 Fig Premature greying of mice lacking Bptf in the melanocytes lineage. **A.** Photographs of mice of the indicated genotypes and post-natal days before onset of hair growth. **B-C.** Photographs of 10 and 14 day-old mice of the indicated genotypes illustrating the characteristics of the first coat with for example variable belly spot and diminished pigmentation of the ears and tail. **D**. Photographs of 21 day-old mice of the indicated genotypes illustrating the greying of the ventral coat. **E**. Genotyping of mouse-tail DNA and DNA from purified melanoblasts detects recombination of the floxed *Bptf* alleles. The upper portion of the figure shows schematically the localisation of the PCR primers with respect to the position of exon 2 of the *Bptf* gene and the inserted LoxP sites (L). The numbers represent the size of the respective PCR products in base pairs. The lower portion of the figure shows the results of the triplex PCR reactions on DNA with the indicated genotypes. The positions of the PCR-products from the WT, Floxed and recombined alleles are indicated. **F.** Photographs of 6 week-old mice that had undergone depilation at 3 weeks of age. The depilated areas are outlined.
(TIF)

S6 Fig Diminished melanoblast proliferation in Bptf-mutant mice. **A-B.** Photographs of representative *Bptf* ^mel+/-^ and *Bptf* ^mel-/-^ E15.5 foetuses in the *Dct*-LacZ background to visualize melanoblasts. Panel A shows the ventral portion and front paw and panel B the trunk and a zoom of representative cells from the trunk region. **C.** Quantification of *Dct*-LacZ-labelled melanoblasts in the indicated regions. An example of the grid used is shown over the limb and paw that is divided into zones (Z). Statistical significance of the difference in cell counts between the Bptf^lox/+^ and Bptf^lox/lox^ embryos was assessed using two-tailed, unpaired Student’s *t*-test (**P < 0.01; ***P < 0.001). N = 4.
(TIF)

S7 Fig Defective differentiation of melanocytes following depilation. **A**. Staining for endogenous Dct and Sox10 10 days after depilation. **B-C**. Staining for endogenous Dct, Pax3, HMB45 and Mitf 10 days after depilation. All scale bars are 50 Scale bars represent 50μm.
(TIF)
