# Supplementary figures and images for: Chromatin-Remodelling Complex NURF Is Essential for Differentiation of Adult Melanocyte Stem Cells
Source: PLoS Genet. 2015 Oct 6;11(10):e1005555. doi: 10.1371/journal.pgen.1005555 (PMC4595011; doi:10.1371/journal.pgen.1005555)

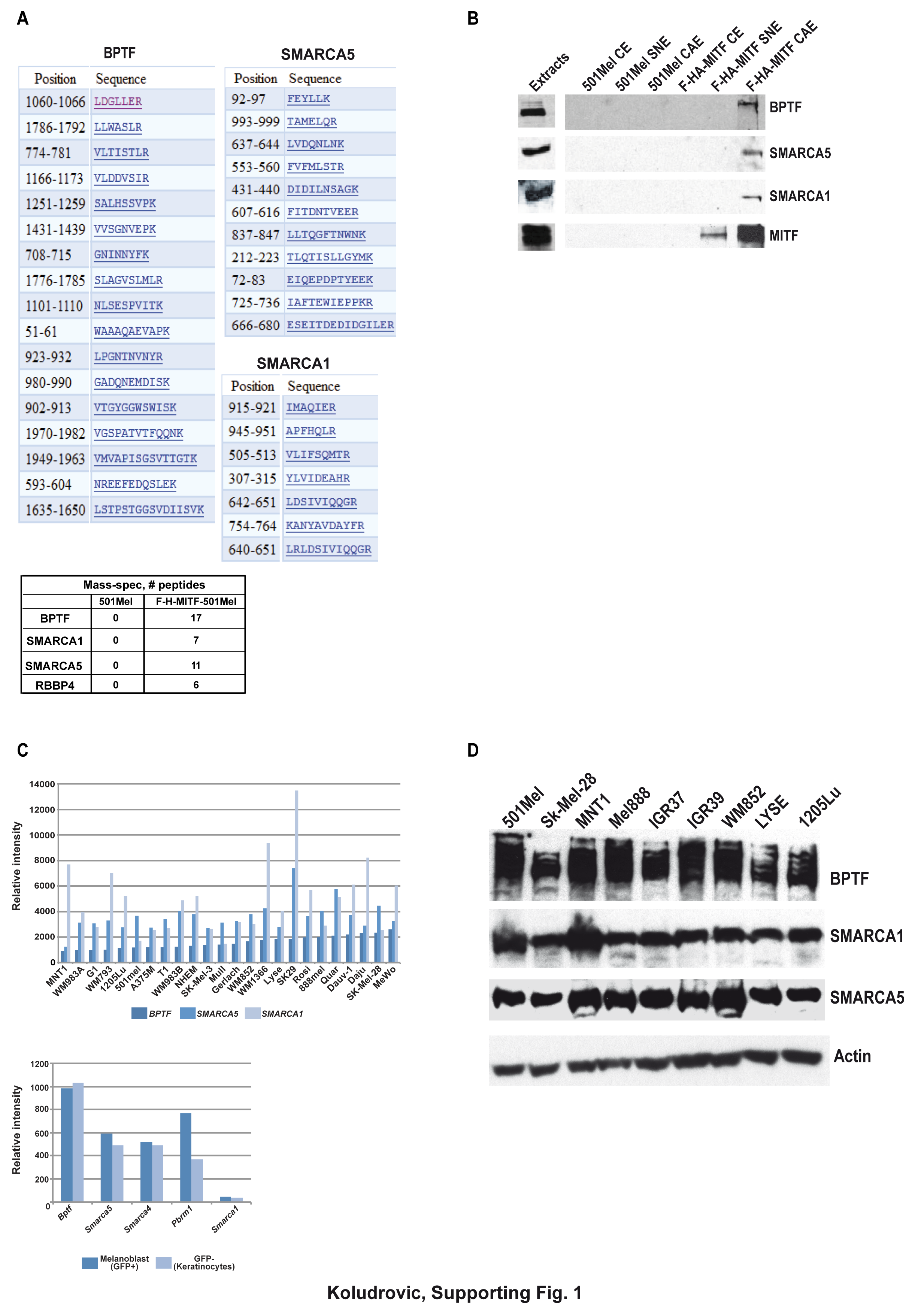

Supplement: S1 Fig — A. The immunoprecipitated material from the soluble nuclear extract (SNE) and chromatin-associated extract (CAE) was analysed by mass-spectrometry. The peptides identified for BPTF, SMARCA1 and SMARCA5 in the chromatin-associated fraction are listed according to their MH+ score. B Immunoblot detection of MITF, BPTF, SMARCA1 and SMARCA5 in FLAG-HA immunoprecipitations of the indicated extracts (CE is cytoplasmic extract) from cells expressing FLAG-HA tagged or native MITF. C. Expression of SMARCA1, SMARCA5 and BPTF in a panel of melanoma cells lines grown in vitro (upper panel) and in developing melanoblasts and keratinocytes (lower panel). D. Total cell extracts were prepared from the indicated cell lines and the presence of the NURF proteins detected by immunoblotting. Note that BPTF is a 400 kDa protein that is extremely sensitive to proteolysis explaining the presence of multiple species. (TIF) [file pgen.1005555.s004.tif]

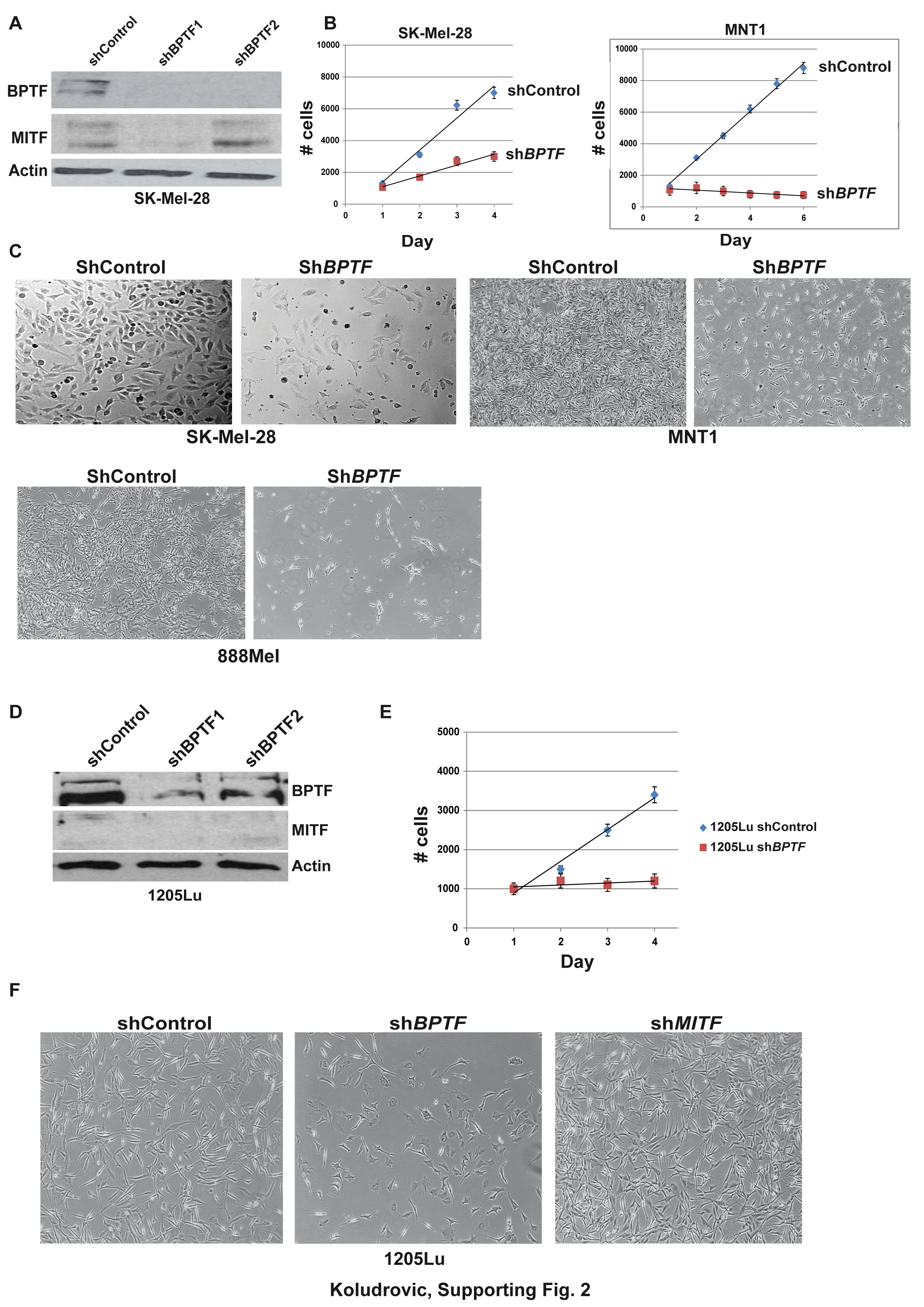

Supplement: S2 Fig — A. Western blot showing knockdown of BPTF and MITF in SK-Mel-28 cells. B. Cell numbers for SK-Mel-28 and MNT1 cells following BPTF knockdown. C. Phase contrast microscopy of SK-Mel-28, MNT1 and 888Mel cells following BPTF knockdown. Magnification X20. D. Western blot showing knockdown of BPTF and absence of MITF in 1205Lu cells. E. Arrested growth of 1205Lu melanoma cells following BPTF knockdown. F. Phase contrast microscopy of 1205Lu cells following BPTF and MITF knockdown. Magnification X20. (TIF) [file pgen.1005555.s005.tif]

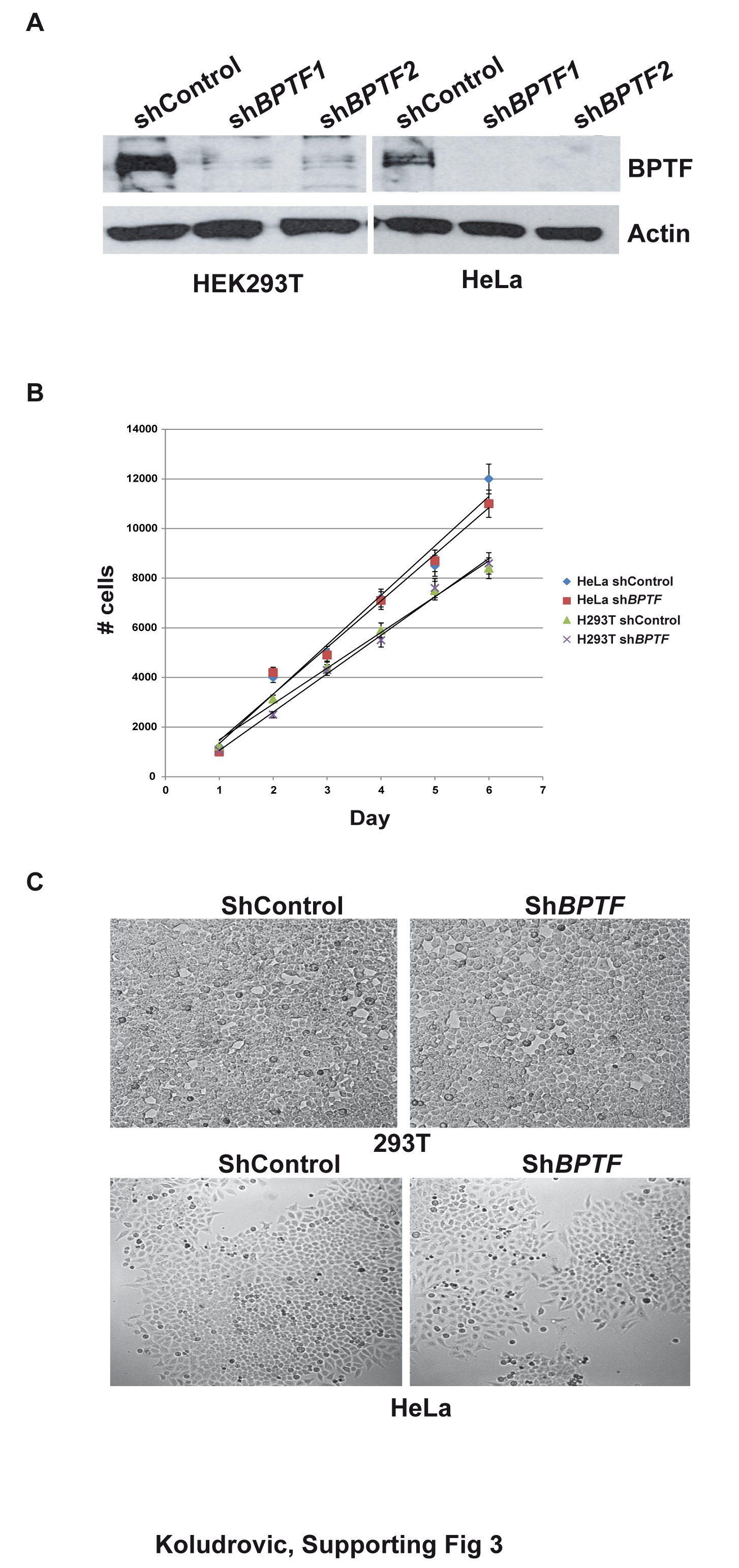

Supplement: S3 Fig — A. Western blot showing knockdown of BPTF in HeLa and HEK293T cells. B. Proliferation of HeLa and HEK293T cells is unaffected by BPTF knockdown. C. Morphology of HeLa and HEK293T cells is unaffected by BPTF knockdown. Magnification X20. (TIF) [file pgen.1005555.s006.tif]

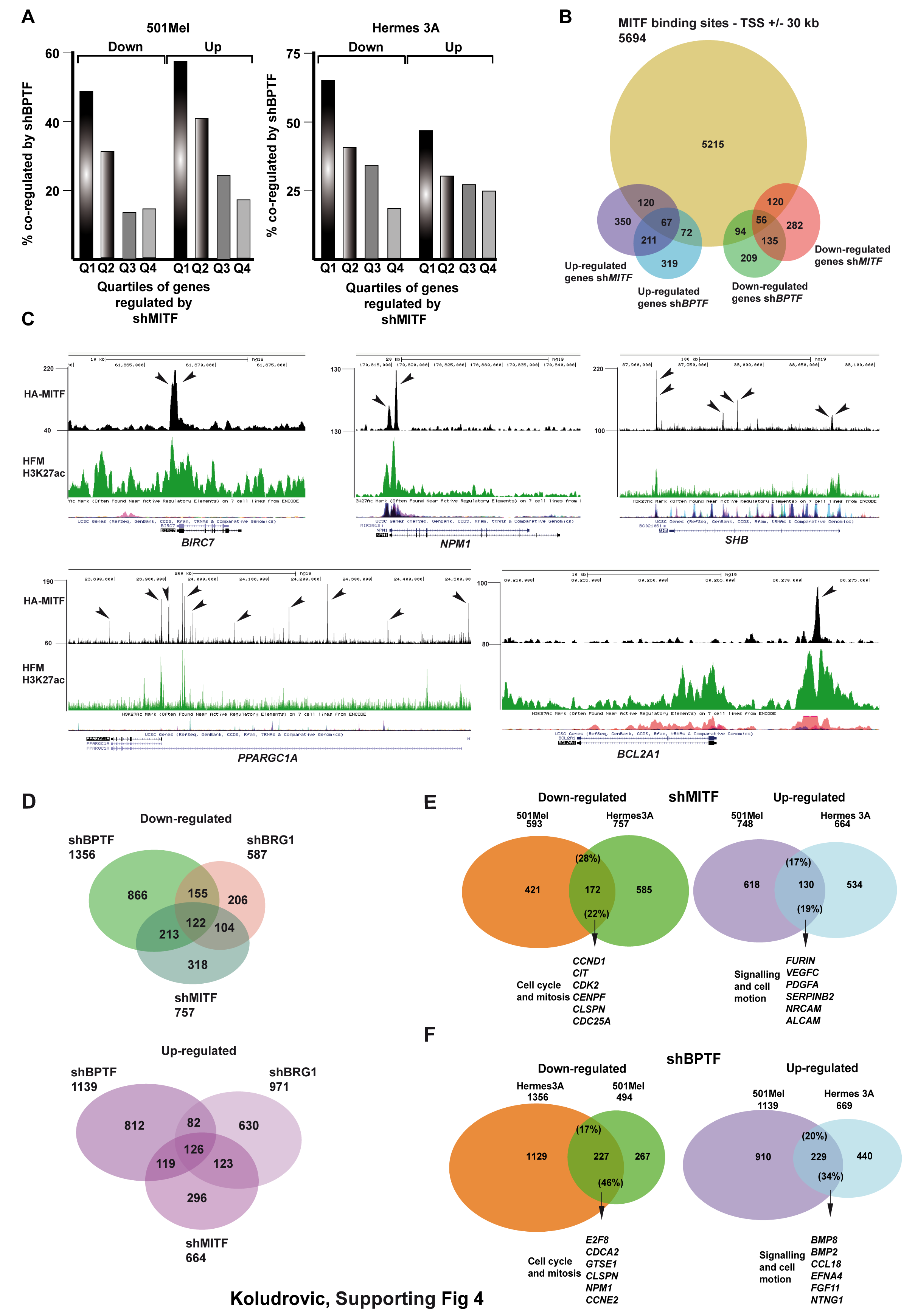

Supplement: S4 Fig — A. The genes regulated by MITF in 501Mel and Hermes 3A cells are divided in quartiles based on their fold change after shMITF silencing. The % of MITF-regulated genes in each quartile co-regulated by BPTF is represented. B. Venn diagrams illustrate the overlap between up and down-regulated genes following shBPTF and shMITF knockdown in 501Mel cells and genes showing an associated MITF-occupied site in ChIP-seq experiments in a +/-30 kb window with respect to the TSS. C. UCSC screenshots of the BIRC7, NPM1, SHB, PPARGC1A and BCL2A1 genes that are associated with MITF-occupied sites and are down-regulated by MITF and BPTF silencing. HA-MITF shows the ChIP-seq track for HA-tagged MITF and arrows indicate representative MITF-occupied sites. HFM indicates the human foreskin melanocyte H3K27ac ChIP-seq track showing promoter and enhancer elements active in the melanocyte lineage. D. Venn diagrams illustrate the overlap between genes up and down-regulated by shBPTF, shMITF and shBRG1 in Hermes 3A cells. E-F Venn diagrams illustrate the overlap between genes up and down-regulated by shBPTF and shMITF in 501Mel and Hermes 3A cells. Several examples of commonly regulated up and down-regulated genes are indicated. (TIF) [file pgen.1005555.s007.tif]

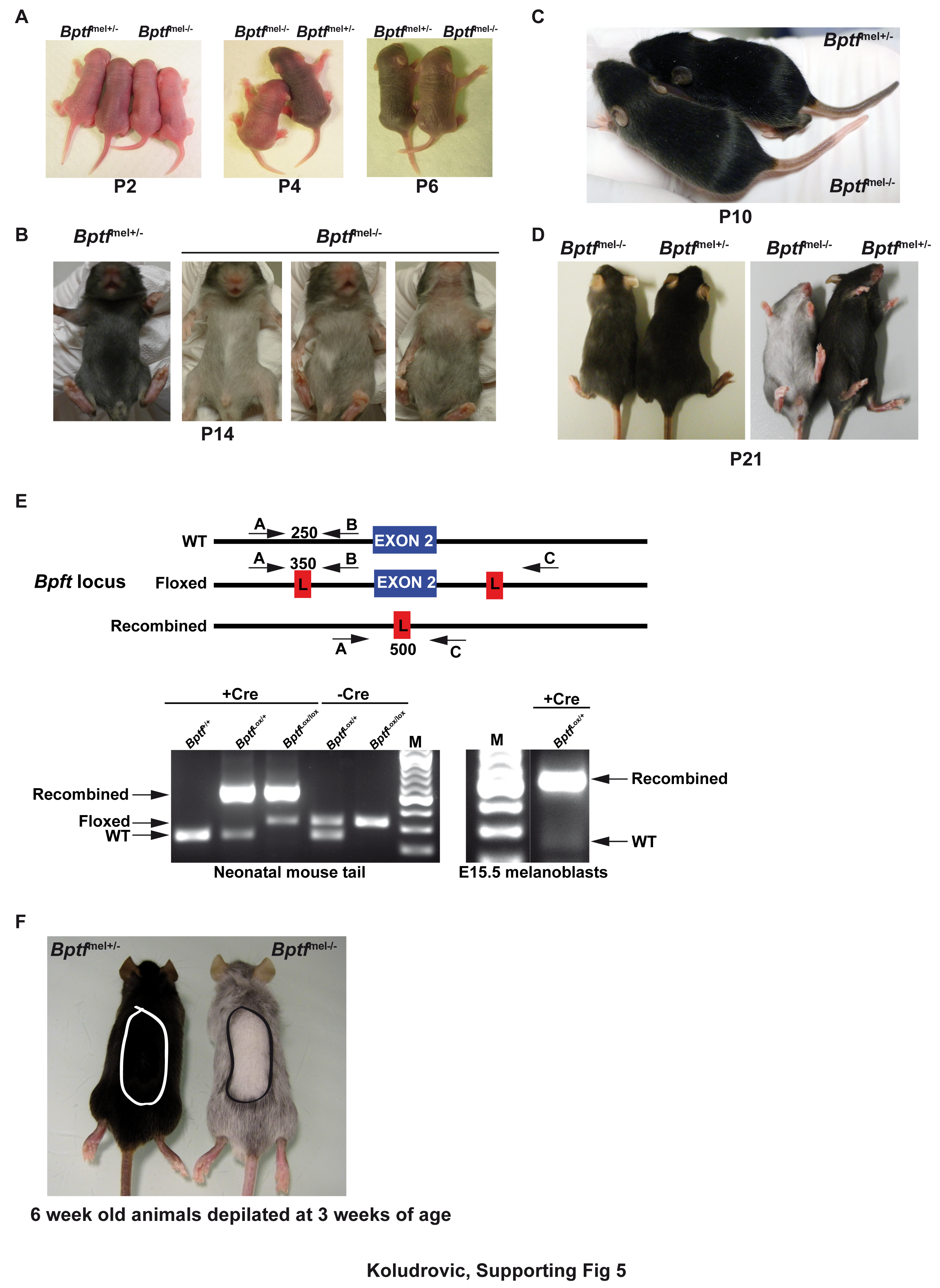

Supplement: S5 Fig — A. Photographs of mice of the indicated genotypes and post-natal days before onset of hair growth. B-C. Photographs of 10 and 14 day-old mice of the indicated genotypes illustrating the characteristics of the first coat with for example variable belly spot and diminished pigmentation of the ears and tail. D. Photographs of 21 day-old mice of the indicated genotypes illustrating the greying of the ventral coat. E. Genotyping of mouse-tail DNA and DNA from purified melanoblasts detects recombination of the floxed Bptf alleles. The upper portion of the figure shows schematically the localisation of the PCR primers with respect to the position of exon 2 of the Bptf gene and the inserted LoxP sites (L). The numbers represent the size of the respective PCR products in base pairs. The lower portion of the figure shows the results of the triplex PCR reactions on DNA with the indicated genotypes. The positions of the PCR-products from the WT, Floxed and recombined alleles are indicated. F. Photographs of 6 week-old mice that had undergone depilation at 3 weeks of age. The depilated areas are outlined. (TIF) [file pgen.1005555.s008.tif]

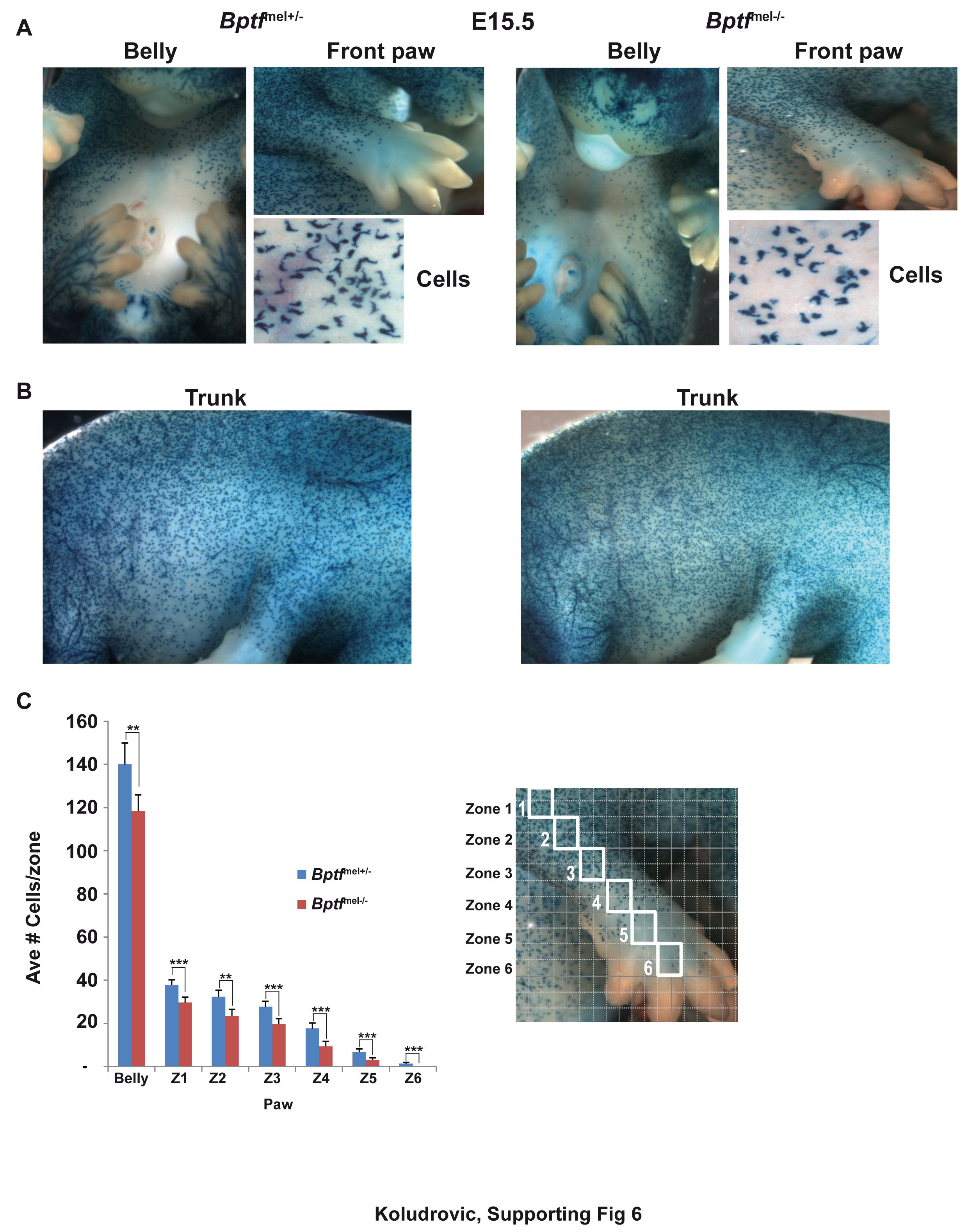

Supplement: S6 Fig — A-B. Photographs of representative Bptf mel+/- and Bptf mel-/- E15.5 foetuses in the Dct-LacZ background to visualize melanoblasts. Panel A shows the ventral portion and front paw and panel B the trunk and a zoom of representative cells from the trunk region. C. Quantification of Dct-LacZ-labelled melanoblasts in the indicated regions. An example of the grid used is shown over the limb and paw that is divided into zones (Z). Statistical significance of the difference in cell counts between the Bptflox/+ and Bptflox/lox embryos was assessed using two-tailed, unpaired Student’s t-test (**P < 0.01; ***P < 0.001). N = 4. (TIF) [file pgen.1005555.s009.tif]

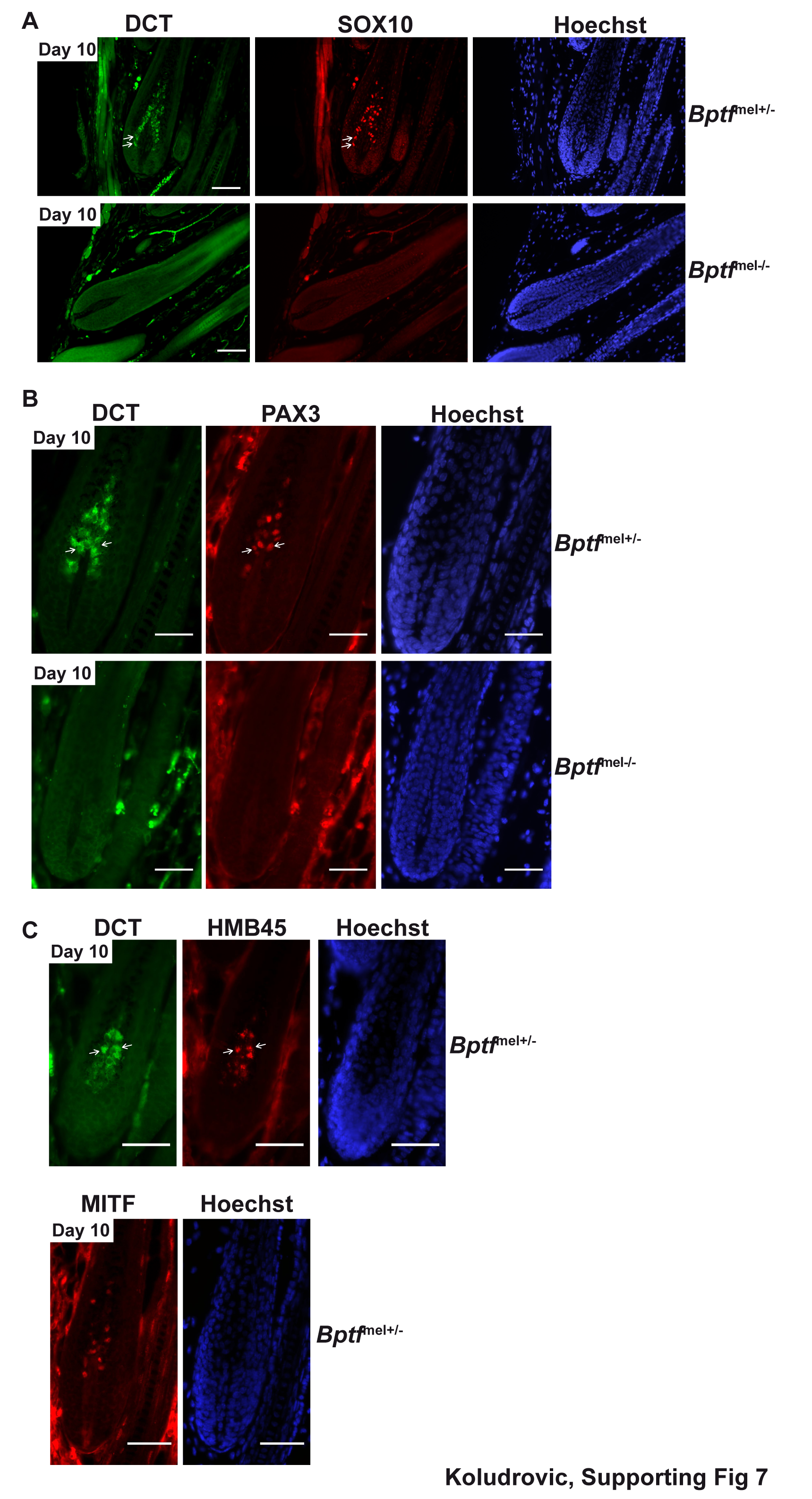

Supplement: S7 Fig — A. Staining for endogenous Dct and Sox10 10 days after depilation. B-C. Staining for endogenous Dct, Pax3, HMB45 and Mitf 10 days after depilation. All scale bars are 50 Scale bars represent 50μm. (TIF) [file pgen.1005555.s010.tif]
